# The Steady State Great Ape? Long Term Isotopic Records Reveal the Effects of Season, Social Rank and Reproductive Status on Bonobo Feeding Behavior

**DOI:** 10.1371/journal.pone.0162091

**Published:** 2016-09-14

**Authors:** Vicky M. Oelze, Pamela Heidi Douglas, Colleen R. Stephens, Martin Surbeck, Verena Behringer, Michael P. Richards, Barbara Fruth, Gottfried Hohmann

**Affiliations:** 1 Max Planck Institute for Evolutionary Anthropology, Department of Primatology, Leipzig, Saxony, Germany; 2 iDiv – German Centre for Integrative Biodiversity Research, Leipzig, Saxony, Germany; 3 Max Planck Institute for Evolutionary Anthropology, Department of Human Evolution, Leipzig, Saxony, Germany; 4 Department of Anthropology, University of British Columbia, Vancouver, British Columbia, Canada; 5 Division of Neurobiology, Ludwig-Maximilians University Munich, Bavaria, Germany; 6 Centre for Research and Conservation, Royal Zoological Society of Antwerp, Antwerp, Flanders, Belgium; University of Otago, NEW ZEALAND

## Abstract

Dietary ecology of extant great apes is known to respond to environmental conditions such as climate and food availability, but also to vary depending on social status and life history characteristics. Bonobos (*Pan paniscus*) live under comparatively steady ecological conditions in the evergreen rainforests of the Congo Basin. Bonobos are an ideal species for investigating influences of sociodemographic and physiological factors, such as female reproductive status, on diet. We investigate the long term dietary pattern in wild but fully habituated bonobos by stable isotope analysis in hair and integrating a variety of long-term sociodemographic information obtained through observations. We analyzed carbon and nitrogen stable isotopes in 432 hair sections obtained from 101 non-invasively collected hair samples. These samples represented the dietary behavior of 23 adult bonobos from 2008 through 2010. By including isotope and crude protein data from plants we could establish an isotope baseline and interpret the results of several general linear mixed models using the predictors climate, sex, social rank, reproductive state of females, adult age and age of infants. We found that low canopy foliage is a useful isotopic tracer for tropical rainforest settings, and consumption of terrestrial herbs best explains the temporal isotope patterns we found in carbon isotope values of bonobo hair. Only the diet of male bonobos was affected by social rank, with lower nitrogen isotope values in low-ranking young males. Female isotope values mainly differed between different stages of reproduction (cycling, pregnancy, lactation). These isotopic differences appear to be related to changes in dietary preference during pregnancy (high protein diet) and lactation (high energy diet), which allow to compensate for different nutritional needs during maternal investment.

## Introduction

Dietary ecology is a key aspect in the study of environmental and social adaptations in primate evolution [1,2]. Recently, stable isotopes have been introduced to the field of primate ecology research as a measure of temporal dietary variation and seasonality [3–5]. Seasonality refers to regular fluctuations in abiotic factors such as climate within the annual cycle, which affect the phenology of plants and consequently the distribution and availability of potential foods. Seasonal scarcity or richness in food resources has strong effects on many aspects of primate ecology, such as the timing of reproduction, mobility, sociality and life history [6]. While some primate species are able to survive in habitats undergoing severe climatic and ecological shifts, others dwell in more stable environments in which foliage or ripe fruit are available all year round [7].

Bonobos (*Pan paniscus*) are a good example of primates adapted to such comparatively stable habitats. Bonobos are found exclusively south of the Congo River in the evergreen rainforests of the Congo Basin. Recently, a comparative isotope study on African great apes by Oelze and coworkers [8] integrated a previously published isotope dataset on wild bonobos [9], which revealed relatively little variation in bonobo stable isotope ratios over time. In particular, variation in nitrogen stable isotopes was lower than in any other analyzed population of apes [8]. Low isotopic variation over time may be explained by the stable climatic characteristics of the Congo Basin [8]. Given the relatively low variability in climatic seasonality in the bonobos’ habitat, it is particularly interesting to study within-group dietary differences that are independent of food abundance. In the present study employing stable isotope analysis in hair, we investigate the temporal dietary pattern in wild bonobos in great detail by integrating a variety of long-term sociodemographic information.

### An isotopic approach to study primate feeding ecology

The stable isotopes of carbon (δ^13^C) and nitrogen (δ^15^N) are increasingly utilized biochemical markers to reconstruct the feeding ecology in extinct and extant primate species [10]. Stable isotope ecology makes use of the fact that “you are what you eat”, as the isotopic characteristics of the main food components are incorporated in consumers’ body tissue with a predictable enrichment caused by isotopic fractionation [11]. Atmospheric carbon enters the biosphere via photosynthesis in green plants. Terrestrial habitats such as tropical rainforests are dominated by C_3_-plants, where variation in δ^13^C values is mainly driven by the so-called “canopy effect” [12,13] and by systematic differences between photosynthetic and non-photosynthetic plant parts [14]. While some primate species, such as baboons, are also known to consume C_4_-plants [4], they are not frequently utilized by great apes, even if C_4_-foods are available in their habitats [15,16].

Nitrogen is predominantly assimilated by plants from the substrate via N_2_-fixing bacteria, which results in differences in the δ^15^N values between locations with different soil properties. With an excess of nitrogen and complex nitrogen pathways, tropical forests are generally enriched in δ^15^N values as compared to temperate forest systems [17]. Within the same location, there are differences in δ^15^N values between the majority of plants utilizing nitrogen from the soil and those plants able to directly assimilate atmospheric nitrogen such as plants of the *Legiminosae* family and some liana species [18,19]. However, δ^15^N values vary most strikingly along the food chain, with the lowest values found in plants, elevated δ^15^N values in primary consumers, and the highest δ^15^N values found in carnivores [20]. This isotopic increase with trophic level make the analysis of δ^15^N values particularly useful in monitoring and quantifying carnivory and insectivory in wild primates [21,22].

A combined study of δ^13^C and δ^15^N patterns in wild animals can provide insight into their feeding niches [23]. Sampling of incremental body tissues such as dental and keratinous tissue can offer additional information on how adaptation to a specific niche varies with time, as seasonal shifts in food availability and/or feeding behavior will be retained in the tissues that developed during the respective time period of dietary change [5,10]. In primatology, two non-invasively collectible sample types have proven particularly useful to reconstruct temporal variation in diet: dung [24] and hair keratin [4,5,9]. While dung rapidly reflects the diet of the previous day(s), hair has the advantage to retain a multi-seasonal dietary signal within a single sample, which can be extracted by segmental sectioning of hair along its growth trajectory [3]. Pioneering work using this hair segmental approach was conducted on domestic cattle [25] and free-ranging African mammals [26–28]. By analyzing tail hair from a group of elephants, Cerling and colleagues [28] could reconstruct the migratory and feeding behavior of an elephant family over multiple years. In great apes, diet changes within the previous five to ten months can be monitored using isotope analysis in individual hair samples. While Western lowland gorillas (*Gorilla gorilla gorilla*) from Gabon have been shown to predominantly shift in δ^13^C values due to varying proportions of herbs versus fruits in their diet, sympatric chimpanzees (*Pan troglodytes troglodytes*) varied significantly in δ^15^N values as they shifted from one species of fruit to another, which had been found to differ in their δ^15^N values [5]. In a recent comparative study including isotope data from all African great ape species, different temporal patterns emerged in hair samples of apes adapted to different environments [8].

### The aims of this study

Here, we present an extensive hair isotope dataset from one group of habituated bonobos over the course of almost two full annual cycles (November 2008 to July 2010). The study extends previous work on bonobo isotope ecology [8,9] by relating measures of isotopic values to information on social and life history parameters. This offers a unique opportunity to assess subtle variation in isotope values within and between individuals that not only consider modest changes in diet but also effects such as age, sex, rank, and reproductive status. Below we formulate predictions how parameters other than food abundance may influence the isotopic signatures in bonobos over time.

#### Consumption of terrestrial herbaceous vegetation

An isotope study on sympatric chimpanzees and gorillas from Gabon found that terrestrial herbaceous vegetation (THV) is a good isotopic marker for primate isotope ecology in habitats dominated by C_3_-plants [5]. We tested this assumption in vegetation samples from the bonobo habitat in Salonga National Park, by adding plant samples to the existing plant dataset from the evergreen rainforest of the Central Cuvette Centrale, Democratic Republic of Congo (DRC), focusing on low canopy plants, such as shrubs and THV. Bonobos are well known for their regular consumption of THV, a particularly protein rich food resource [29]. We hypothesize that if bonobo isotopic data varies in response to season, then the seasonal effect will be larger in the δ^13^C values due to the shifting proportions of high canopy ripe fruit versus low canopy THV in their diet.

#### Social factors influencing diet

Besides seasonal variation in food availability affecting all individuals in the same way, age, sex, and social status are also known to influence the dietary repertoire of free-ranging primates. For instance, investigating stable isotopes of habituated Western chimpanzees (*Pan troglodytes verus*), Fahy and colleagues [21] were able to show that males consumed significantly more meat than females. Also, certain males were observed to have higher success rates in hunting than others, which resulted in higher isotope values due to the larger amount and higher frequency of meat consumed. In the context of chimpanzee hunting, both the physical and the social dominance of certain males may play a role in the ability to successfully hunt and monopolize prey [30]. In bonobos, a different dietary pattern has been described via stable isotope analysis. While almost all individuals of one group were similar in their dietary signature, low-ranking adolescent males had significantly lower δ^15^N values, suggesting that they were excluded from certain high trophic level food resources [9]. We used long-term isotopic data to test to what extend differences in isotope values are related to social rank, sex and age. Based on previous work on bonobo isotope ecology, we predict that female bonobos do not differ isotopically from males. However, low-ranking adolescent males are expected to be depleted in their nitrogen stable isotope values as they commonly are excluded from high trophic level foods, e.g. vertebrate meat.

#### Reproductive status of females

In addition to the ecological and social factors influencing the feeding behavior of wild primates, there are several physical conditions that need to be considered when studying primate feeding ecology by means of isotopes. Only recently, Loudon and colleagues [31] emphasized the importance of understanding the isotopic variation caused by age and sex differences within a primate community to make inferences on primate feeding behavior. Nutritional stress is known to influence the isotopic signatures of animal body tissues [32,33]. Gestation and particularly lactation are considered to be physiologically and energetically demanding periods in female primate life [34,35]. During these phases, dietary energy is not only invested in the individual’s own metabolism, but also in maternal and fetal protein synthesis and milk production. Few studies have considered the effect of maternal investment during pregnancy and lactation on female isotope ratios, and these are limited to captive animals and modern humans [36–38]. A study of pregnant women showed that while the carbon isotopes remained the same, the nitrogen signatures declined significantly during gestation although the dietary input was assumed to be relatively constant [36]. Reitsema and colleagues [37] pointed out that the diversity of agricultural food items which primates are exposed to in zoos could mask some physiological shifts in stable isotope ratios. This is probably also the case for humans living in industrial societies [36,38]. The data from wild female bonobos presented here can start filling this gap.

The long-term isotopic dataset allows us to relate isotopic values from females to different reproductive states, and to control how isotope signatures of females during different reproductive stages relate to overall changes that may reflect temporal differences in food abundance. Our hypothesis is that isotope ratios of female bonobos will differ from each other during the different phases of reproduction (cycling, pregnancy, lactation).

## Materials and Methods

This study was carried out at the field site of LuiKotale (02° 45.610’S, 20° 22.723’E), near Salonga National Park, DRC [39]. Climate parameters including minimum and maximum temperatures in °C and precipitation amounts in mm were measured daily at the field site. We combined previously measured bonobo hair stable isotope values (n = 95 from 39 hair samples) dating from 2008 to 2009 [8,9] with a substantial new isotope dataset (n = 337) obtained from 62 bonobo hair samples collected in 2010 (S1 Table). The resulting dataset of a total of 432 hair section measurements is the largest isotope dataset to date on a group of extant great apes. All new hair samples were retrieved by an experienced tree climber from night nests of free-ranging bonobos of the Bompusa community in LuiKotale. Individuals building nests were identified during nest-to-nest follows and trees were climbed to pick hair samples after the bonobos left the nest site. Hair sample collection in the wild was strictly non-invasive and thus not subject to recommendations of the Weatherall report (The Use of Non-Human Primates in Research). Hairs samples were stored dry in pergamin envelopes and shipped to Germany adhered to the Convention on International Trade in Endangered Species of Wild Fauna and Flora (CITES), including CITES documents (N/Ref. 00651/ICCN/ DG/ADG/KBY/2011).

As previous vegetation samples from Salonga forest were biased toward high canopy foods (fruits), additional plant food items (n = 37) were collected opportunistically, with a focus on low canopy foods between 2010 and 2013, and stored dry on silica. We also included data on plant crude protein from previous studies on bonobo feeding ecology [40] in our analyses of these plants (S2 Table).

The community’s dominance hierarchy was based on behavioral interactions observed between December 2007 and July 2009, as described in detail elsewhere [41]. While in early 2009 the study community consisted of 35 individuals [9], several individuals disappeared from the community in 2009 (n = 2) and 2010 (n = 5). Consequently, the calculated linear dominance hierarchy within the community was adjusted after each disappearance event for statistical analyses. Information on life histories of individual bonobos, including estimated or known birth year, sex, age of infant, and month/year of disappearance were gathered from the long-term database of the LuiKotale Bonobo Research project. The categories for female reproductive status (cycling, pregnant or lactating) were based on data available on infant birth dates (see S1 Table and details below).

### Isotope analysis

Hair sample preparation followed the procedure outlined in Oelze [3]. Hairs were washed in a chloroform/methanol solution (2:1 v/v) in a rotator overnight to remove lipids and external contaminants [42]. Dried hair strands were transferred to a microscope working space and sorted for hairs with roots in the telogen stage (pale yellowish root bulbs) to reduce growth cycle error [43] as much as possible. Furthermore, all short and thin hairs were excluded to avoid unintentional sampling of infant individuals [3,9], leaving per hair sample, on average, 12 hairs of 6–7 cm length of equal thickness and color. These samples were then aligned at their roots and sectioned with a scalpel into 5 and 10 mm sections as weight allowed. Finally, each hair section (≥ 0.2 mg) was transferred into tin capsules for isotopic measurement.

All plant items were dried in an oven at 50°C for several days, homogenized with a pestle and mortar, and weighed into tin capsules (~ 2 mg) for duplicate isotopic measurement. All measurements were performed parallel to the IAEA standards CH6, CH7, N1 and N2 as well as several internal standard materials in a Flash EA 2112 (Thermo-Finnigan^®^, Bremen, Germany) coupled to a DeltaXP mass spectrometer (Thermo-Finnigan^®^, Bremen, Germany) at the Max Planck Institute for Evolutionary Anthropology in Leipzig, Germany. The stable isotope ratios of carbon and nitrogen are expressed as the ratio of ^13^C/^12^C and ^15^N/^14^N ratios using the delta (δ) notation in parts per thousand or per mil (‰) relative to the international standard materials Vienna PeeDee Belemite (vPDB) and atmospheric N_2_ (AIR). Measurement error calculated from these standard materials is less than 0.2 ‰ (1σ) for the δ^13^C and δ^15^N values. To ensure analytical quality, we excluded nine hair isotope data points with atomic C/N ratios outside the acceptable 2.6 to 3.8 range [42]. We excluded all data points obtained from bulk hair samples reported in the previous work by Oelze and coworkers [9]. Due to a lack of hair growth rates for the genus *Pan*, we considered *Homo sapiens* hair growth rates of 10 mm for 28 days [44] in the subsequent data analyses.

### Statistical analyses

We used R (version 3.1.0, [45]] to conduct statistical analyses. We employed several general linear mixed models (GLMM) with Gaussian error structure using the lmer-function [46] on the responses δ^15^N and δ^13^C to test effects of various predictors on plant and bonobo hair isotope data.

#### Plant models

We used a Spearman correlation to investigate the relationship between the results of the elemental analyses in plants (n = 70) and previous phytochemical analyses on protein content (n = 43) from the same plant species and parts [40]. We calculated crude protein content of plants from %N values measured in plants by multiplying with the factor 6.25 ([40], and see S2 Table). Using a GLMM we tested for differences in protein content among different categories of plants (herb, shrub, tree fruit, and tree non-food). Additionally, we ran two GLMMs looking for possible isotopic differences in δ^15^N and δ^13^C values among plant categories. We took multiple measurements of some plant species into account by including ‘species’ (n = 47) as a random effect in each model.

#### Bonobo models

We ran GLMMs separately for the response variables δ^15^N and δ^13^C measured in 434 bonobo hair sections of 23 known individuals. We included the climatic data to represent the predictor ‘season’ and carefully evaluated the inclusion of other main predictors individual age, social rank, and sex, as well as the reproductive status of females and the age of dependent infants in order to test several hypotheses at once. In these models we included the random slope term of ‘temp_timelag_’ (see below) within ‘individual’, and included ‘hair ID’ (sample) and ‘individual’ as random effects to account for the fact that multiple measurements were taken per hair sample and also per individual [47].

#### Calculating time lags in the climate variable

To test how the isotope ratios in bonobo hair sections were influenced by season across several years, we focused on climatic variation that has been shown to influence tree fruiting and thus may also affect the bonobos’ feeding repertoire with an uncertain time lag. Phenology of fruit trees is often related to climatic factors such as temperature, rainfall, and insolation, but the specifics of these relationships seem to vary among locations, species and seasons [48–50]. Given that temperature varied over the year at LuiKotale, whereas precipitation did not reveal any clear-cut dry or rainy seasons (see S1 Fig), we used daily mean temperature data, which we smoothed using locally weighted polynomial regression via the lowess function in R [51] to obtain a seasonal pattern across the study period. We considered different time lags for the effect of temperature (‘temp_timelag_’) on the timing of fruit ripening, but we did not consider longer time lags (> 80 days), which would be related to flower production or pollination conditions in previous months or years. We ran 66 models (including all other predictors as see below) with different time lags between 14 and 80 days and compared their Akaike Information Criteria (AIC in the following, see also S2A and S2B Fig). As temperature was significant (or at least a strong trend) in each of these 66 test-models, we could select the model with the best AIC, which suggested the best time lags of 52 days for the δ^13^C model and 28 days for the δ^15^N model. We used temperature data that incorporated these best time lags in the subsequent models with which we tested the other predictors. Given that dietary nitrogen and carbon are metabolized and excreted separately, different response times in the two isotope systems can be expected. This has been reported for other tissue types and a range of species, although no consistent pattern could yet be described and some authors report longer turnover rates for nitrogen than for carbon [45,46]. To obtain a likelihood ratio test of significance for the effect of temperature in bonobo hair with a time lag we averaged the chi-squared values from the 66 models and added one degree of freedom, as temp_timelag_ represents two terms (temperature and time lag) in the model.

#### Integrating individual age, rank, sex and reproductive status

We predict that the isotope values may vary in relation to social ranks and probably between ages. In a previous study, the effects of rank and age were significant in bonobo males [9]. In this dataset a correlation between social rank and age revealed the relationship is significant (R = 0.865, N = 21, p < 0.001) so we only included rank as a fixed effect in the models. The factors sex and reproductive status were combined as one factor (hereafter ‘sex/reproductive status’) as we assumed there could be isotopic differences between males and females per se, but also between females cycling, pregnant and lactating. As we thought the effect of rank might differ depending on the sex/reproductive status of an individual, we first tested for their interaction in each model. Likelihood ratio tests between models with and without the interaction revealed the interaction between rank and sex/reproductive status was significant in the δ^15^N values (χ^2^ = 13.633, df = 3, p < 0.004), but not in the δ^13^C values (χ^2^ = 3.978, df = 3, p = 0.264).

The interaction term was thus included in the δ^15^N model, and in the following rank was always tested in an interaction with sex/reproductive status. This interaction could be dropped from the δ^13^C model, thus social rank could be tested as a single main effect. To test if individual age is actually a better predictor than social rank we compared the AICs of the full δ^13^C and δ^15^N models with the fixed effects of sex and rank in an interaction with two alternative models: a) substituting rank with individual age; and b) including both the age and the rank term. The AICs of these different models were the same in the δ^15^N model (less than 0.6 ΔAIC), suggesting that the effect of individual age and rank are interchangeable in respect to δ^15^N values. For δ^13^C values however, both models including age as predictor revealed smaller AICs (80.065 for both and 80.052 for age only) than the model only including social rank (AIC = 83.672).

#### Reproductive status of females

In a post-hoc approach we tested the effect of infant age (–days prior to birth, 0day = estimated day of birth, +days after birth) on the mother’s δ^13^C and δ^15^N values in a subset of females which were pregnant or had dependent offspring (< 3 years old), and for which the date of birth of the infant could be estimated with a precision of ±15 days (7 females, N = 163, see S1 Table). We used an average gestation length of 224 days in bonobos [52] to distinguish between cycling and pregnant females. The fixed effect ‘infant age’ was z-transformed and included in an interaction with reproductive status of females as a linear as well as a squared term (‘infant age^2^’) to allow for different model slope patterns for female hair isotope ratios before and after parturition. The significance of the effect of infant age was established using likelihood ratio tests comparing the full model to a null model excluding all terms relating to infant age. We conducted model diagnostics as outlined below. Controls of model stability (see below) suggested one female (Gwen) was influential in the δ^15^N model, but did not clearly alter the overall results.

#### Model diagnostics

On all the above models we conducted model diagnostics by visually inspecting qq-plots and the residuals plotted against fitted values, which both confirmed normally distributed and homogeneous residuals. In the full models we selected those with the best temp_timelag_ and the two alternative models with the highest and lowest temp_timelag_ [14 and 80 days) for diagnostics. We also tested Variance Inflation Factors from the results of a standard linear model, which excluded any interactions, polynomials, random effects and random slopes and found no evidence for collinearity. We then tested model stability by excluding levels of random effects [hair sample, individual), one at a time and comparing the results to the original models. These tests suggested that two females, Olga and Uma, had some influence on the stability of the full δ^15^N model, but did not influence the overall results. We obtained confidence intervals and slopes for the different sexes and reproductive stages by dummy coding and re-leveling the factor sex/reproductive status.

## Results and Discussion

### Plant isotopes and THV consumption

We found that previous measurements on plant crude protein (%dry mass) are positively correlated with %N measures obtained in this study during stable isotope analysis (Spearman correlation: R = 0.83, N = 43, p < 0.001). This is not surprising as phytochemical analyses used an elemental analyzer system for determination of nitrogen content. These %N values were converted into crude protein estimations using a conversion factor of 6.25 [40]. We applied the same equation to our %N data and found that estimates of crude protein content differs significantly between the different food categories as defined in S1 Table (χ^2^ = 9.871, df = 4, p = 0.042), with the highest protein content in terrestrial herbaceous vegetation and the lowest in fruits, which is well in line with previous nutritional research on plant foods consumed by *Pan* species [29]. We suggest calculations of crude protein based on %N could be considered more frequently in dietary reconstructions by means of stable isotopes, e.g. in stable isotope mixing models [53]. If we assume the protein fraction of the diet has a stronger effect on isotope ratios in body proteins such as hair [54], the relevance of specific food items could be weighted by their crude protein content in such models.

While the plant δ^15^N values of different plant categories (herb, shrub, tree fruit, tree non-food) did not differ from each other (χ^2^ = 2.807, df = 4, p = 0.590), differences between plant categories were highly significant in terms of δ^13^C values (χ^2^ = 31.348, df = 4, p < 0.0001). We found the lowest δ^13^C values in herbs and shrubs and the highest values in fruits of trees. This suggests a vertical stratigraphy in δ^13^C values within the forest canopy [12], but probably also differences between plant parts [14] as seen between fruits and non-fruit items of the same tree species (see S2 Table). Bonobos feeding on these plant foods in different proportions would be expected to show low variation in their hair δ^15^N values, but higher variation in their δ^13^C values, particularly if they switch between high canopy fruits and low canopy foliage such as THV.

THV is an important component in bonobo diet and this protein rich terrestrial vegetation is abundant in forested habitats inhabited by them [29,55]. The regular THV consumption by bonobos has been hypothesized to be a key parameter explaining the high levels of female gregariousness in this species [56] with implication for sex-differences in cooperation and other aspects of their social lives [57,58].

### Seasonal patterns

**Fig 1:**
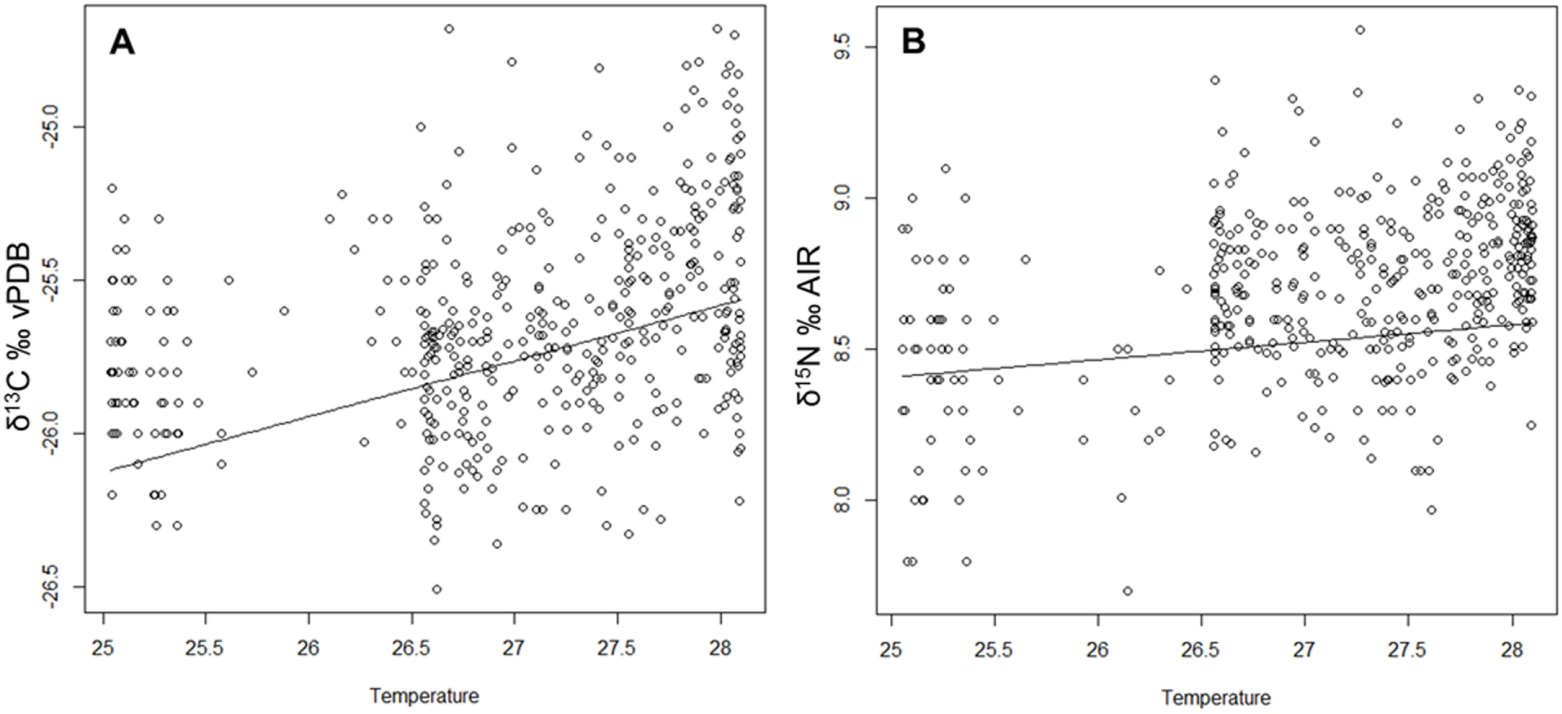
GLMM model results suggested significant increases in δ^13^C (A) and δ^15^N (B) values with increase in temperature (°C), representing season. This isotopic increase is more pronounced in δ^13^C values than in δ^15^N values.

When including the climate data into our statistical analyses we found that the effect of temperature with a time lag of 52 and 28 days respectively was significant in the δ^13^C model (mean weighted χ^2^ = 26.071, df = 8, p = 0.0010) and in the δ^15^N model (mean weighted χ^2^ = 29.791, df = 8, p = 0.0002). Our results show that δ^13^C and δ^15^N values in bonobo hair significantly increased as temperature increased (see Fig 1A and 1B). However, these isotopic increases were admittedly very small; our model estimate revealed with 1°C increase in temperature a 0.11‰ increase in δ^13^C values and an increase of 0.06 ‰ in δ^15^N values. Our results suggest the effect of temporal variation in response to temperature (season) is overall subtle, yet more pronounced in δ^13^C values than in δ^15^N values, which is well in line with the stronger variation in plant δ^13^C values and the more homogenous plant δ^15^N values in the Salonga forest. Based on the plant δ^13^C baseline, we conclude that low δ^13^C values in bonobos indicate the consumption of higher proportions of low canopy foods. In this respect the bonobo isotope pattern is similar to that of western lowland gorillas (*Gorilla gorilla gorilla*) from Loango, Gabon, which varied significantly in their hair δ^13^C values over time, but not in their δ^15^N values [5]. The hair δ^13^C values of Loango gorillas ranged from -27.1 ‰ to -24.5 ‰, whereas the bonobo isotope values we report here ranged from -26.5 ‰ to -24.7 ‰. This suggests that the dietary shifts are less pronounced in bonobos as compared to Western lowland gorillas. We expected to find a dietary pattern with low variation in a habitat with little seasonal variation. The comparatively constant environmental conditions within this area of the Congo Basin distinguish the bonobos’ feeding ecology from other great ape species in Africa, particularly in respect to δ^15^N signatures [8]. It remains to be tested whether the same pattern can be detected in other *Pan* populations within the Congo Basin.

### Effects of social rank and sex

**Fig 2:**
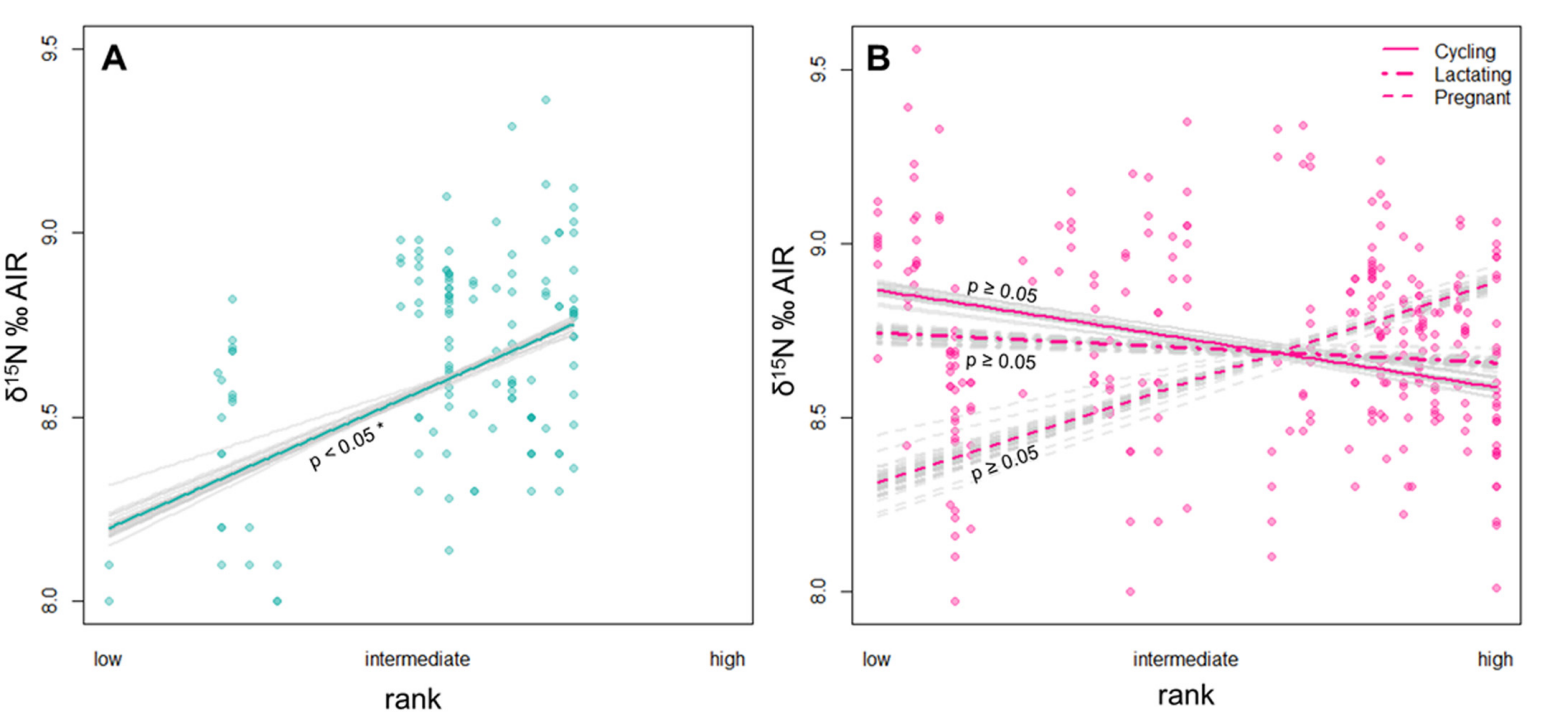
Model results for the effect of social dominance rank on the δ^15^N values in male (A) and female (B) bonobos. Rank had a significant effect on δ^15^N values in males, but not in females. Model stability is indicated by the grey alternative model lines, suggesting some influence of single hair samples and individuals which did not influence our overall results.

In this new extended dataset on bonobo hair isotopes the effect of sex/reproductive status interacting with social rank was significant in δ^15^N values (χ^2^ = 13.63, df = 3, p < 0.004). This indicates that the effect of rank on δ^15^N values and thus probably the access to ^15^N-enriched food items (e.g. animal protein) is different between males and/or females with the different reproductive status. Fig 2A and 2B show that the effect of rank on the bonobos’ isotope signatures differs between males and females: in females, the regression lines do not differ significantly from zero, regardless of reproductive status, indicating no differences in δ^15^N values due to social rank. Low-ranking males, however, have significantly lower δ^15^N values than high-ranking males and also than females.

Sex and social rank is known to influence the access to certain foods in *Pan* species. Limited resources such as meat or certain fruit may be shared with non-kin to maintain or strengthen social bonds with coalition partners [58–60]. Indeed adolescent male bonobos are often excluded from meat sharing events [58,61], and although the first isotope study did not reveal evidence for faunivory at LuiKotale, less frequent meat consumption may be a plausible explanation for depleted δ^15^N values in low-ranking adolescent males.

In the δ^13^C values we could test sex/reproductive status separately from social rank. While the effect of sex/reproductive status was significant (χ^2^ = 16.056, df = 3, p = 0.0011), social rank did not predict bonobo δ^13^C values (χ^2^ = 0.1638, df = 1, p = 0.686). However, for alternative δ^13^C-models, including age instead of social rank and both age and social rank revealed slightly lower AICs than the original model only comprising social rank. This suggested better model fits for models considering the effect of age in δ^13^C values, but our approach did not allow for testing this effect further for significance. The differences in δ^13^C values between females with different reproductive statuses and males can be interpreted without the interacting effect of social dominance rank. We discuss our findings in more detail below as the effect appears to be linked to the reproductive status of females and not to sex in general.

### Reproductive status of females

The isotope ecology at LuiKotale, with comparatively low seasonality, allows for investigating possible variation that is not only linked to resource availability and dietary choice but also to differences in physiological conditions among individuals with different reproductive states. We expected cycling females without dependent offspring would be similar to males, while pregnant females or females with dependent offspring would differ isotopically from other adult community members.

#### Carbon reflecting food selection

**Fig 3:**
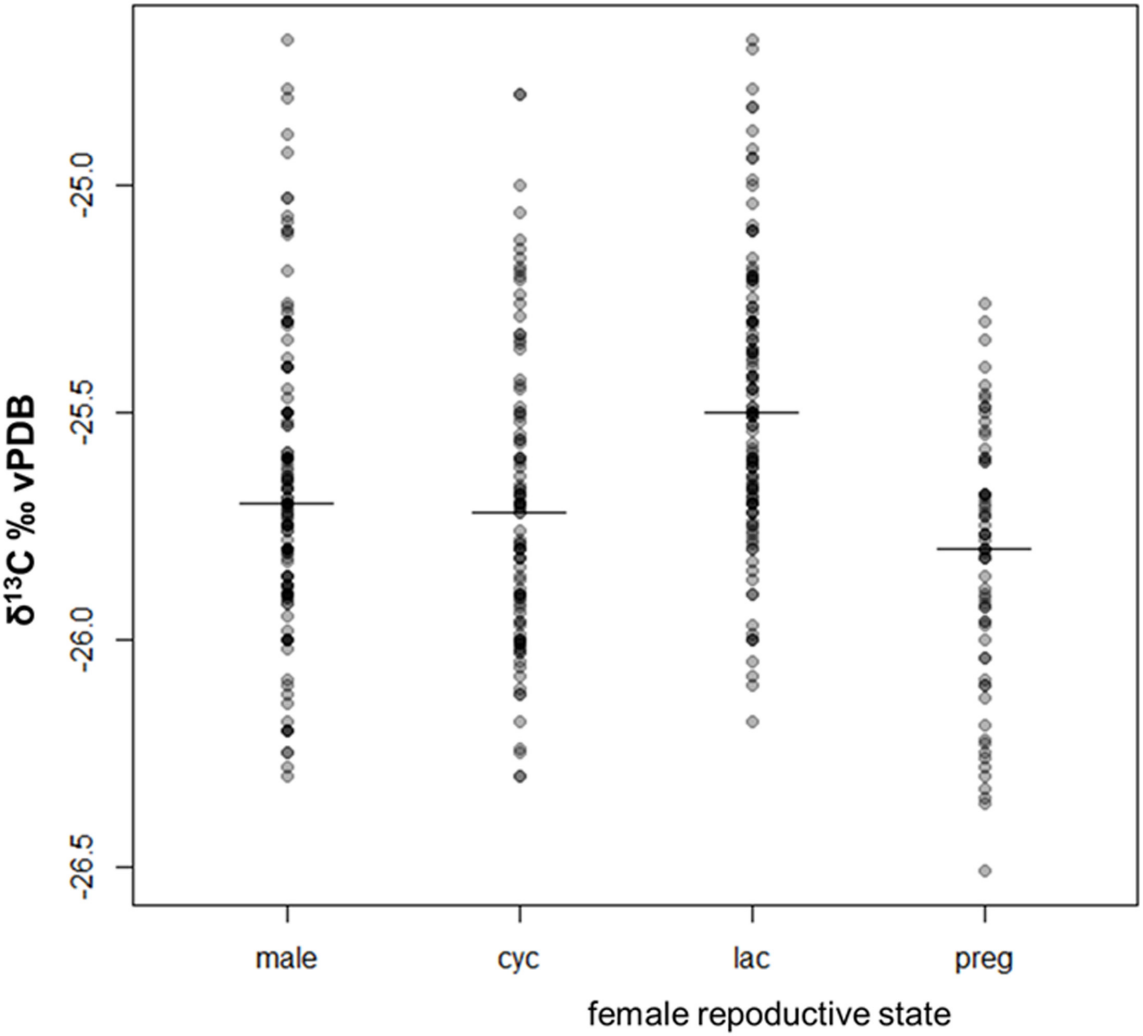
δ^13^C values and medians for bonobo males, cycling (cyc), lactating (lac) and pregnant females (preg). According to our model the differences between males and females with different reproductive statuses are significant in δ^13^C values, with the highest values in lactating females. This relationship could not be tested in the δ^15^N values given the significant interaction of sex/reproductive state with social dominance rank.

As stated above, the bonobos’ δ^13^C values clearly differed between males and females with different reproductive statuses (χ^2^ = 16.06, df = 3, p < 0.002). Although overall variation in δ^13^C values is small (~1.5 ‰) lactating females had significantly higher δ^13^C values than other individuals (Fig 3). While males and cycling females without dependent offspring were very similar in δ^13^C values (their medians are almost identical), pregnant females had the lowest δ^13^C values (Fig 3). For the δ^13^C values we predicted the isotope ratios should be slightly higher in lactating females and decrease again as the infant becomes less dependent, as it has been shown in a study on captive langurs (*Trachypithecus francoisi*) [37]. However, in the post-hoc δ^13^C model, which included the age of the respective dependent offspring, the effect of infant age and the squared term of infant age (‘infant age²’) interacting with reproductive status was not significant (χ^2^ = 1.134, df = 2, p = 0.567). Overall, the largest differences in δ^13^C values of female bonobos can be observed between gestation and lactation, both of which are physiologically demanding phases for mammalian females [62]. Pregnancy resulted in lower δ^13^C values, while lactation tended to result in enriched δ^13^C values. Previous isotope studies on female primates, including humans, found contradictory isotopic patterns related to pregnancy and lactation in δ^13^C values (36–38), which may suggest a weak effect of physiological constitution on δ^13^C values in the body.

If physiology does not cause the observed pattern found in female bonobos, dietary preference may explain the shifted δ^13^C values of pregnant and lactating females. During pregnancy, protein demands increase in the mother due to the protein synthesis of maternal and fetal tissue [63] and female bonobos may respond to these demands by increased feeding on high protein foods such as THV. Above we presented that THV has particularly low δ^13^C values which could explain low δ^13^C values in females investing in fetal development. During the energetically demanding period of lactation [34], female bonobos may preferentially consume high caloric fruits, which have high δ^13^C values in this and other forest habitats [5,8,24].

#### Nitrogen reflecting maternal investment

**Fig 4:**
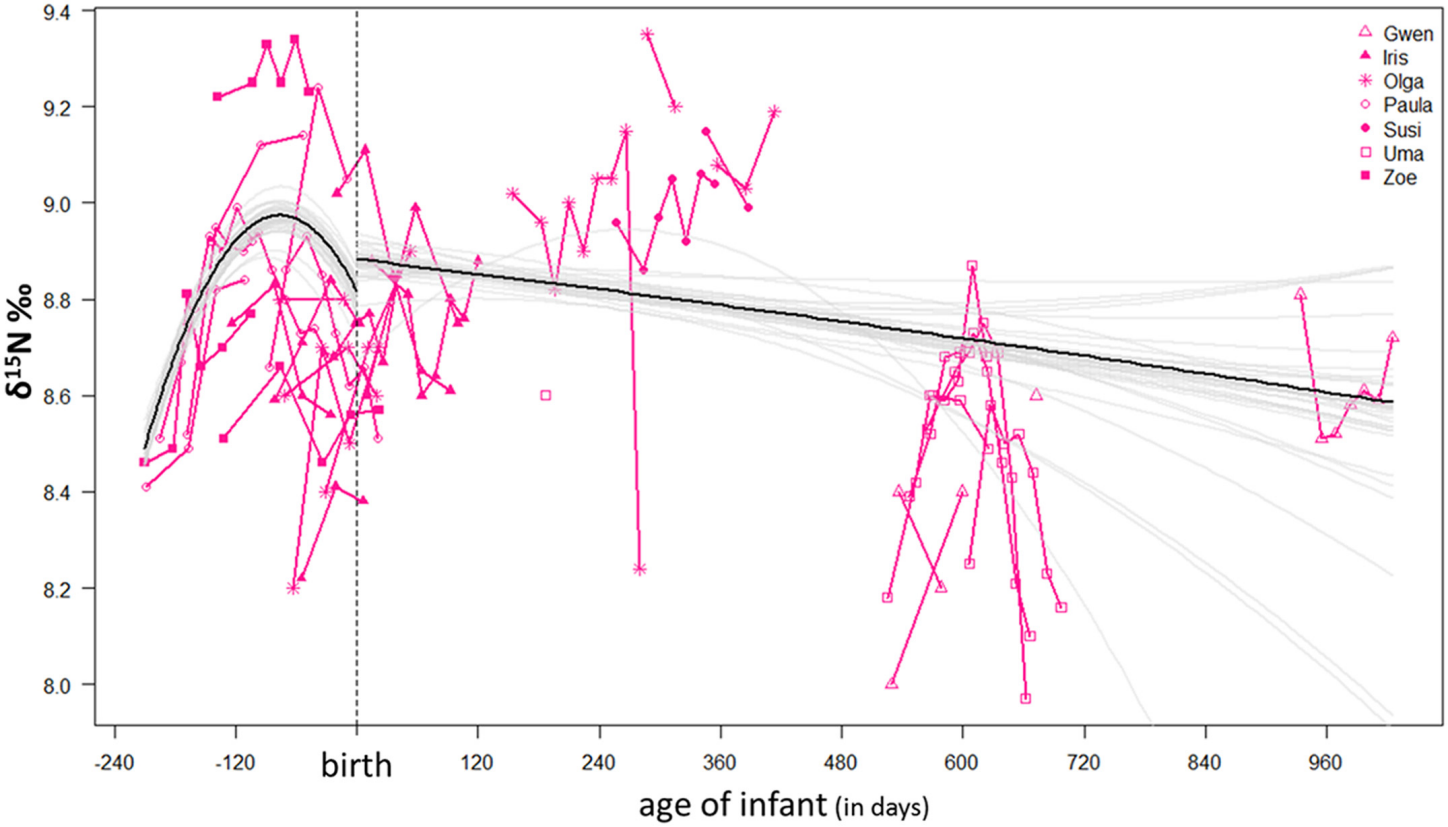
Post-hoc model on female bonobo reproductive status and age of infant. The relationship between the variation in δ^15^N values and reproductive status interacting with age of offspring was clearly significant, suggesting that δ^15^N values change during pregnancy and within the lactation period with increasing age of infant. The model stability is indicated as alternative model lines (in gray) which fit well during pregnancy but also illustrate that it is unstable for lactating females, particularly as infants become increasingly independent from their mothers.

For nitrogen, we predicted depleted δ^15^N values during pregnancy as reported for humans [36,38] and probably also depleted δ^15^N values during the early phase of lactation [37]. In δ^15^N values the effect of infant age and infant age² interacting with reproductive status was significant (χ² = 17.98, df = 4, p < 0.002), suggesting the δ^15^N-pattern differs between pregnancy and the time after parturition. Fig 4 illustrates that female bonobo δ^15^N values were enriched during pregnancy and decreased again towards the time of parturition, represented by an upside u-shape. This pattern contradicts what Fuller and colleagues [38] described in δ^15^N values for a sample of ten pregnant women from the USA. However, we assume it is unlikely that the δ^15^N pattern we observe in wild bonobos is connected to food resource utilization. First, plant food δ^15^N values are remarkably homogeneous in the Salonga forest habitat (see above). Secondly, consumption of ^15^N-enriched foods such as meat is overall rather rare and hunting is limited to small-bodied prey [9,64,65]. Thirdly, as we controlled for seasonal variation in the model these differences should not be related to a short-term dietary shift such as the availability of particularly ^15^N-enriched fruit or a hunting season. Hence, this pattern should be of physiological nature and may reflect the mobilization and metabolism of body-own proteins during gestation. Hominoids, including humans and great apes, are considered capital breeders, which refers to our ability to store fat reserves to cope with seasonal constraints in food resources. This strategy allows females to cycle year round in absence of fertilization, and reproduction is not limited to seasonal peaks in food availability. In capital breeders offspring can remain dependent over several years and thus multiple seasons [66,67]. However, if parous females physically invest their own body capital (energy and protein) in the offspring, this would indeed affect their protein balance [32]. Our findings suggest caution when considering small scale isotopic variation over time, as they apparently can be related to maternal investment and not to feeding behavior per se (Fig 4).

Finally, the model predicts the δ^15^N values are highest in the time after parturition and then decrease with increasing age of the infant. Here, one potential explanation of slightly increased values after parturition could be the ingestion of placenta tissue immediately following the birth event. Only very recently, Douglas [68] observed placentophagia in this community of wild bonobos and described how placenta tissue was shared with females attending the birth event. However, as this event takes place high up in the forest canopy the absolute amounts of ingested placental tissue are impossible to assess. Nevertheless, this high trophic level dietary resource should be considered in this context as it may be important for female nutrition after parturition [69].

## Conclusions

Our long-term isotope dataset on free-ranging habituated bonobos is the most comprehensive of its kind to date and the first allowing for testing the relationships between environmental, social and demographic factors, and stable isotope ratios in hair. Based on our plant analyses we conclude that low canopy foliage such as THV can be considered a useful isotopic tracer in tropical forest settings to reconstruct feeding niches. THV is particularly low in δ^13^C values, rich in crude protein and thus its consumption should be well detectable in hair protein. We were able to show that social dominance rank does indeed affect individual access to high trophic level food resources in male bonobos, but not in females. In females, our results suggest the diet mainly differs between individuals in different phases of reproduction. If our interpretation of female bonobo isotope patterns is suitable, the δ^13^C values of female primates are mainly driven by food choice, while the δ^15^N values in these capital breeders may be influenced additionally by the physiological demands of reproduction. This assumption should be tested in other primate species in the future. Our findings may find increased consideration in future ecological research employing stable isotope analysis in wild and captive capital breeders.

## Supporting Information

S1 Fig Climatic variation measured in LuiKotale, DRC. (TIF)

S2 Fig **A and B**: AICs obtained from 66 models each for the δ^13^C values and δ^15^N values with different time lags (14 to 80 days) of temperature (indirectly representing season and plant phenology/fruiting) having an effect on the variation in bonobo hair isotopic ratios. Points under the dashed line represent models with a ΔAIC ≤2, suggesting a similarly high level of support for those models.
(TIF)

S1 Table Bonobo hair section stable isotope data including hair sampling date and estimated mean hair section date and all further information on individuals (indiv.) including age (estimated birth year), social rank, sex, age of infant (inf. age) in days prior/after parturition, reproductive state of females (rep. state), and also disappearance date (month/year) for individuals who were not seen in the study community during later phases of the study period. (PDF)

S2 Table Plant stable isotope data for the different plant food type categories in the LuiKotale bonobo habitat (*crude protein data from [40]). (PDF)

## References

[1] Hohmann G, Robbins MM, Boesch C, editors. Feeding ecology in apes and other primates: ecological, physical, and behavioral aspects. Cambridge, Eng.; New York: Cambridge University Press; 2006.

[2] Ungar PS, Sponheimer M. The diets of early hominins. Science. 2011; 334:190–193. 10.1126/science.1207701 21998380

[3] Oelze VM. Reconstructing temporal variation in great ape diets: a methodological framework for isotope analyses in non-invasively collected hair. Am J Primatol. 2016; 10.1002/ajp.22497 26530015

[4] Codron D, Lee-Thorp JA, Sponheimer M, de Ruiter D, Codron J. Inter- and intrahabitat dietary variability of chacma baboons (*Papio ursinus*) in South African savannas based on fecal delta C-13, delta N-15, and %N. Am J Phys Anthropol. 2006;129:204–214. 16247809 10.1002/ajpa.20253

[5] Oelze VM, Head JS, Robbins MM, Richards M, Boesch C. Niche differentiation and dietary seasonality among sympatric gorillas and chimpanzees in Loango National Park (Gabon) revealed by stable isotope analysis. J Hum Evol. 2014;66:95–106. 10.1016/j.jhevol.2013.10.003 24373257

[6] van Schaik CP, Terborgh JW, Wright SJ. The phenology of tropical forests: Adaptive significance and consequences for primary consumers. Annu Rev Ecol Syst. 1993;24:353–377.

[7] Brockman DK, van Schaik CP. Seasonality in Primates: Studies of living and extinct human and non-human primates. Cambridge: Cambridge University Press; 2005.

[8] Oelze VM, Fahy GE, Hohmann G, Robbins MM, Leinert V, Lee K, et al Comparative Isotope Ecolog of African Great Apes. J Hum Evol.Accepted manuscript (18/8/2016). 10.1016/j.jhevol.2016.08.007 27886808

[9] Oelze VM, Fuller BT, Richards MP, Fruth B, Surbeck M, Hublin J-J, et al Exploring the contribution and significance of animal protein in the diet of bonobos by stable isotope ratio analysis of hair. Proc Natl Acad Sci. 2011;108:9792–9797. 10.1073/pnas.1018502108 21628564 PMC3116404

[10] Crowley BE, Reitsema LJ, Oelze VM, Sponheimer M. Advances in primate stable isotope ecology—Achievements and future prospects. Am J Primatol. 2016; 10.1002/ajp.22510 26683892

[11] Kohn MJ. You are what you eat. Science. 1999;283:335–336. 9925492 10.1126/science.283.5400.335

[12] Medina E, Minchin P. Stratification of d^13^C values of leaves in Amazonian rain forests. Oecologia. 1980;45:377–378. 28309567 10.1007/BF00540209

[13] van der Merwe NJ, Medina E. The canopy effect, carbon isotope ratios and foodwebs in Amazonia. J Archaeol Sci. 1991;18:249–259.

[14] Cernusak LA, Tcherkez G, Keitel C, Cornwell WK, Santiago LS, Knohl A, et al Why are non-photosynthetic tissues generally ^13^C enriched compared with leaves in C_3_ plants? Review and synthesis of current hypotheses. Funct Plant Biol. 2009;36:199–213. 10.1071/FP08216 32688639

[15] Sponheimer M, Passey BH, de Ruiter DJ, Guatelli-Steinberg D, Cerling TE, Lee-Thorp JA. Isotopic evidence for dietary variability in the early hominin *Paranthropus robustus*. Science. 2006;314:980–982. 17095699 10.1126/science.1133827

[16] Schoeninger MJ, Moore J, Sept JM. Subsistence strategies of two “savanna” chimpanzee populations: The stable isotope evidence. Am J Primatol. 1999;49:297–314. 10553959 10.1002/(SICI)1098-2345(199912)49:4<297::AID-AJP2>3.0.CO;2-N

[17] Martinelli LA, Piccolo MC, Townsend AR, Vitousek PM, Cuevas E, McDowell W, et al Nitrogen stable isotopic composition of leaves and soil: Tropical versus temperate forests. Biogeochemistry. 1999;46:45–65.

[18] Gehring C, Vlek PLG. Limitations of the ^15^N natural abundance method for estimating biological nitrogen fixation in Amazonian forest legumes. Basic Appl Ecol. 2004;5:567–580.

[19] Peterson BJ, Fry B. Stable Isotopes in Ecosystem Studies. Annu Rev Ecol Syst. 1987;18:293–320.

[20] Minagawa M, Wada E. Stepwise enrichment of ^15^N along food chains: further evidence and the relation between ^15^N and animal age. Geochim Cosmochim Acta. 1984;48:1135–40.

[21] Fahy GE, Richards M, Riedel J, Hublin J-J, Boesch C. Stable isotope evidence of meat eating and hunting specialization in adult male chimpanzees. Proc Natl Acad Sci. 2013;110:5829–5833. 10.1073/pnas.1221991110 23530185 PMC3625252

[22] Schoeninger MJ, Iwaniec UT, Glander KE. Stable isotope ratios indicate diet and habitat use in New World monkeys. Am J Phys Anthropol. 1997;103:69–83. 9185952 10.1002/(SICI)1096-8644(199705)103:1<69::AID-AJPA5>3.0.CO;2-8

[23] Wolf N, Carleton SA, Martínez de Rio C. Ten years of experimental animal isotope ecology. Funct Ecol. 2009;23:17–26.

[24] Blumenthal SA, Chritz KL, Rothman JM, Cerling TE. Detecting intraannual dietary variability in wild mountain gorillas by stable isotope analysis of feces. Proc Natl Acad Sci. 2012;109:21277–21282. 10.1073/pnas.1215782109 23236160 PMC3535629

[25] Schwertl M, Auerswald K, Schnyder H. Reconstruction of the isotopic history of animal diets by hair segmental analysis. Rapid Commun Mass Spectrom. 2003;17:1312–1318. 12811754 10.1002/rcm.1042

[26] Cerling TE, Viehl K. Seasonal diet changes of the forest hog (*Hylochoerus meinertzhageni Thomas*) based on the carbon isotopic composition of hair. Afr J Ecol. 2004;42:88–92.

[27] Cerling TE, Wittemyer G, Ehleringer JR, Remien CH, Douglas-Hamilton I. History of Animals using Isotope Records (HAIR): A 6-year dietary history of one family of African elephants. Proc Natl Acad Sci. 2009;106:8093–8100. 10.1073/pnas.0902192106 19365077 PMC2688856

[28] Cerling TE, Wittemyer G, Rasmussen HB, Vollrath F, Cerling CE, Robinson TJ, et al Stable isotopes in elephant hair document migration patterns and diet changes. Proc Natl Acad Sci. 2006;103:371–373. 16407164 10.1073/pnas.0509606102 PMC1326175

[29] Malenky RK, Stiles EW. Distribution of terrestrial herbaceous vegetation and its consumption by Pan paniscus in the Lomako Forest, Zaire. Am J Primatol. 1991;23:153–169. 10.1002/ajp.1350230303 31952402

[30] Boesch C, Boesch H. Hunting behavior of wild chimpanzees in the Taï National Park. Am J Phys Anthropol. 1989;78:547–573. 2540662 10.1002/ajpa.1330780410

[31] Loudon JE, Grobler P, Sponheimer M, Moyer K, Lorenz JG, Turner TR. Using the stable carbon and nitrogen isotope compositions of vervet monkeys (*Chlorocebus pygerythrus*) to examine questions in ethnoprimatology. PLOS ONE. 2014; 9:e100758 10.1371/journal.pone.0100758 25010211 PMC4091945

[32] Deschner T, Fuller BT, Oelze VM, Boesch C, Hublin J-J, Mundry R, et al Identification of energy consumption and nutritional stress by isotopic and elemental analysis of urine in bonobos (*Pan paniscus*). Rapid Commun Mass Spectrom. 2012;26:69–77. 10.1002/rcm.5312 22215580

[33] Vogel ER, Knott CD, Crowley BE, Blakely MD, Larsen MD, Dominy NJ. Bornean orangutans on the brink of protein bankruptcy. Biol Lett. 2012;8:333–336. 10.1098/rsbl.2011.1040 22171019 PMC3367743

[34] Lee PC. Nutrition, fertility and maternal investment in primates. J Zool. 1987;213:409–422.

[35] Thompson ME, Muller MN, Wrangham RW. The energetics of lactation and the return to fecundity in wild chimpanzees. Behav Ecol. 2012; 23:1234–1241.

[36] Fuller BT, Fuller JL, Sage NE, Harris DA, O’Connell TC, Hedges REM. Nitrogen balance and delta15N: why you’re not what you eat during pregnancy. Rapid Commun Mass Spectrom. 2004;18:2889–2896. 15517531 10.1002/rcm.1708

[37] Reitsema LJ. Introducing fecal stable isotope analysis in primate weaning studies. Am J Primatol. 2012;74:926–939. 10.1002/ajp.22045 22729669

[38] Fuller BT, Fuller JL, Harris DA, Hedges REM. Detection of breastfeeding and weaning in modern human infants with carbon and nitrogen stable isotope ratios. Am J Phys Anthropol. 2006;129:279–293. 16261548 10.1002/ajpa.20249

[39] Hohmann G, Fruth B. Lui Kotale- A new site for field research on bonobos in the Salonga National Park. Pan Afr News. 2003;10:25–27.

[40] Hohmann G, Potts K, N’Guessan A, Fowler A, Mundry R, Ganzhorn JU, et al Plant foods consumed by *Pan*: Exploring the variation of nutritional ecology across Africa. Am J Phys Anthropol. 2010;141:476–485. 10.1002/ajpa.21168 19918996

[41] Surbeck M, Hohmann G. Intersexual dominance relationships and the influence of leverage on the outcome of conflicts in wild bonobos (*Pan paniscus*). Behav Ecol Sociobiol. 2013;67:1767–1780.

[42] O’Connell TC, Hedges REM, Healey MA, Simpson AHRW. Isotopic comparison of hair, nail and bone: modern analyses. J Archaeol Sci. 2001;28: 1247–1255.

[43] Williams LJ, White CD, Longstaffe FJ. Improving stable isotopic interpretations made from human hair through reduction of growth cycle error. Am J Phys Anthropol. 2011;145:125–136. 10.1002/ajpa.21479 21312184

[44] Tobin DJ. The biogenesis and growth of hair In: Tobin DJ, editor. Hair in toxycology: An important bio-monitor. Cambridge: The Royal Society of Chemistry; 2005 p. 3–33.

[45] R Development Core Team. R: A language and environment for statistical computing. Vienna, Austria: R Foundation for Statistical Computing; 2013.

[46] Baayen RH. Analyzing linguistic data. Cambridge: Cambridge University Press; 2008.

[47] Mundry R, Oelze VM. Who is who matters—The effects of pseudoreplication in stable isotope analyses. Am J Primatol. 2016; 10.1002/ajp.22499 26581644

[48] Anderson DP, Nordheim EV, Moermond TC, Gone Bi ZB, Boesch C. Factors influencing tree phenology in Taï National Park, Côte d’Ivoire. Biotropica. 2005;37:631–640.

[49] Tutin CEG, Fernandez M. Relationships between minimum temperature and fruit production in some tropical forest trees in Gabon. J Trop Ecol. 1993;9:241–248.

[50] Walsh RPD. Climate In: Richards PW, editor. The tropical rain forest: an ecological study. Cambridge: Cambridge University Press; 1996 p. 159–205.

[51] Cleveland WS. LOWESS: A program for smoothing scatterplots by robust locally weighted regression. Am Stat. 1981;38: 261–269.

[52] Drews B, Harmann LM, Beehler LL, Bell B, Drews RF, Hildebrandt TB. Ultrasonographic monitoring of fetal development in unrestrained bonobos (*Pan paniscus*) at the Milwaukee County Zoo. Zoo Biol. 2011;30:241–253. 10.1002/zoo.20304 20073051

[53] Hopkins JB, Ferguson JM. Estimating the diets of animals using stable isotopes and a comprehensive bayesian mixing model. PLOS ONE. 2012;7:e28478 10.1371/journal.pone.0028478 22235246 PMC3250396

[54] Ambrose SH. Isotopic analysis of palaeodiets: methodological and interpretative considerations In: Sandford MK, editor. Investigations of ancient human tissue: chemical analyses in anthropology. Langhorne, Pennsylvannia: Gordon and Breach; 1993 p. 59–130.

[55] Malenky RK, Wrangham RW. A quantitative comparison of terrestrial herbaceous food consumption by *Pan paniscus* in the Lomako Forest, Zaire, and *Pan troglodytes* in the Kibale Forest, Uganda. Am J Primatol. 1994;32:1–12. 10.1002/ajp.1350320102 31936906

[56] Chapman CA, White FJ, Wrangham RW. Party size in chimpanzees and bonobos In: Wrangham RW, McGrew WC, de Waal FBM, Heltne P, editors. Chimpanzee cultures. Cambridge and London: Harvard University Press; 1994 p. 41–58.

[57] Hohmann G, Fowler A, Sommer V, Ortmann S. Frugivory and gregariousness of Salonga bonobos and Gashake chimpanzees: The influence of abundance and nutritional quality of fruit In: Hohmann G, Robbins M, Boesch C, editors. Feeding ecology in apes and other primates—ecological, physiological and behavioural aspects. Cambridge: Cambridge University Press; 2006 p. 123–160.

[58] Fruth B, Hohmann G. How bonobos handle hunts and harvests: why share food? In: Boesch C, Hohmann G, Marchant L, editors. Behavioural Diversity in Chimpanzees and Bonobos. Cambridge: Cambridge University Press; 2002 p. 231–243.

[59] Hockings KJ, Humle T, Anderson JR, Biro D, Sousa C, Ohashi G, et al Chimpanzees share forbidden fruit. PLOS ONE. 2007;2:e886 17849015 10.1371/journal.pone.0000886 PMC1964537

[60] McGrew WC. Dominance status, food sharing, and reproductive success in chimpanzees In: Wiessner P, Schiefenhoevel W, editors. Food and the status quest: An interdisciplinary perspective. Oxford: Berghan Books; 1996 p. 39–45.

[61] Hohmann G, Fruth B. Field observations on meat sharing among bonobos (*Pan paniscus*). Folia Primatol. 1993;60:225–229.

[62] McClure PA. The energetics of reproduction and life histories of cricetine rodents In: Loudon A, Racey PA, editors. The reproductive energy in mammals. Oxford: Oxford University Press; 1987 p. 241–258.

[63] Kalhan SC. Protein metabolism in pregnancy. Am J Clin Nutr. 2000;71(5 Suppl):1249s –1255s. 10799398 10.1093/ajcn/71.5.1249s

[64] Hohmann G, Fruth B. New records on prey capture and meat eating by bonobos at Lui Kotale, Salonga National Park, Democratic Republic of Congo. Folia Primatol. 2008;79:103–110. 17975315 10.1159/000110679

[65] Surbeck M, Fowler A, Deimel C, Hohmann G. Evidence for the consumption of arboreal, diurnal primates by bonobos (*Pan paniscus*). Am J Primatol. 2009;71:171–174. 10.1002/ajp.20634 19058132

[66] Brockmann D, Van Schaik CP. Seasonality and repoductive function In: Brockmann D, Van Schaik CP, editors. Seasonality in primates: Studies of living and extinct human and non-human primates. Cambridge: Cambridge University Press; 2005 p. 269–305.

[67] Stephens PA, Boyd IL, McNamara JM, Houston AI. Capital breeding and income breeding: their meaning, measurement, and worth. Ecology. 2009;90:2057–2067. 19739368 10.1890/08-1369.1

[68] Douglas PH. Female sociality during the daytime birth of a wild bonobo at Luikotale, Democratic Republic of the Congo. Primates. 2014;55:533–542. 10.1007/s10329-014-0436-0 25007717

[69] Kristala MB, DiPirrob JM, Thompsonc AC. Placentophagia in humans and nonhuman mammals: Causes and consequences. Ecol Food Nutr. 2012;51:177–197. 10.1080/03670244.2012.661325 22632059

