# Supplementary figures and images for: The Steady State Great Ape? Long Term Isotopic Records Reveal the Effects of Season, Social Rank and Reproductive Status on Bonobo Feeding Behavior
Source: PLoS One. 2016 Sep 14;11(9):e0162091. doi: 10.1371/journal.pone.0162091 (PMC5023189; doi:10.1371/journal.pone.0162091)

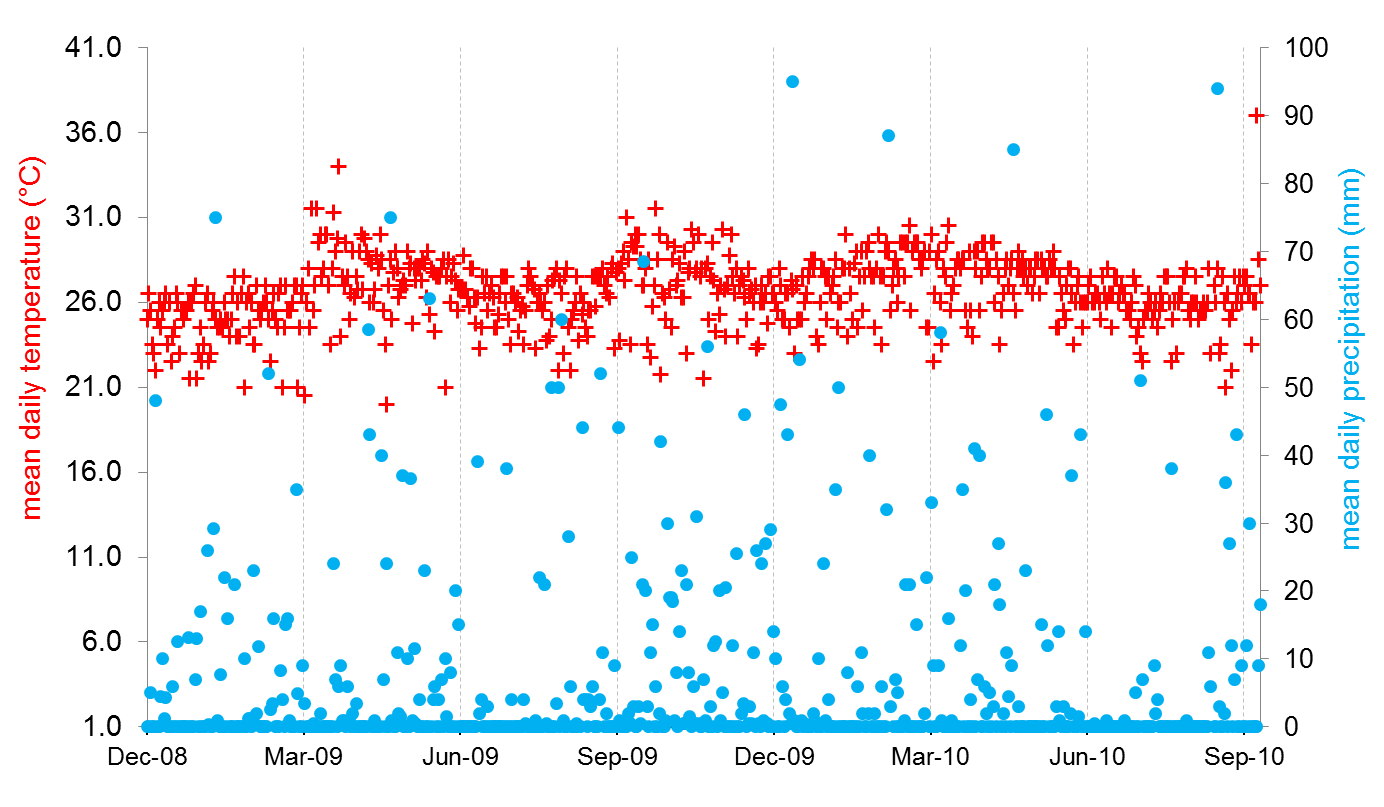

Supplement: S1 Fig — (TIF) [file pone.0162091.s001.tif]

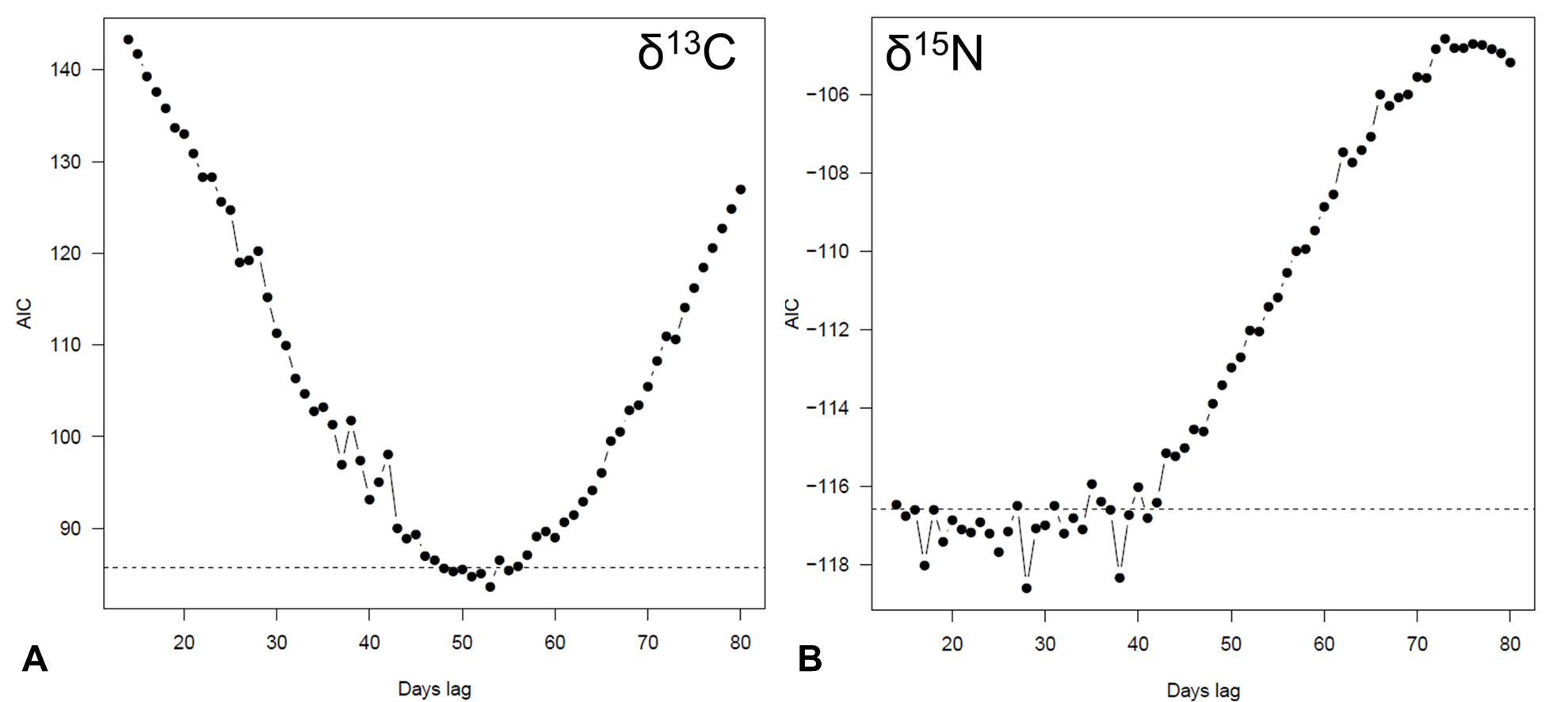

Supplement: S2 Fig — A and B: AICs obtained from 66 models each for the δ13C values and δ15N values with different time lags (14 to 80 days) of temperature (indirectly representing season and plant phenology/fruiting) having an effect on the variation in bonobo hair isotopic ratios. Points under the dashed line represent models with a ΔAIC ≤2, suggesting a similarly high level of support for those models. (TIF) [file pone.0162091.s002.tif]
