# Supplementary material for: The Steady State Great Ape? Long Term Isotopic Records Reveal the Effects of Season, Social Rank and Reproductive Status on Bonobo Feeding Behavior
Source: PLoS One. 2016 Sep 14;11(9):e0162091. doi: 10.1371/journal.pone.0162091 (PMC5023189; doi:10.1371/journal.pone.0162091)
Supplement: S1 Table — (PDF) [file pone.0162091.s003.pdf]

hair ID (lab no.)	indiv.	sex	rank*	birth year	rep. state	age infant (days)	disapear day	disappear month	disappear year	sampling date	hair section date	$\delta^{13}C$ ‰ vPDB	$\delta^{15}N$ ‰ AIR
13405	Eva	f	4	1967	cyc	1690	20	11	2009	1-Sep-09	18-Aug-09	-25.6	8.3
13405	Eva	f	4	1967	cyc	1648	20	11	2009	1-Sep-09	7-Jul-09	-25.6	8.5
14098	Eva	f	4	1967	lac	1639	20	11	2009	19-Jul-09	28-Jun-09	-25.3	8.8
14098	Eva	f	4	1967	lac	1584	20	11	2009	20-Jul-09	4-May-09	-25.5	8.9
21708	Gwen	f	14	1994	lac	1025	NA	NA	NA	13-Jul-10	6-Jul-10	-25.5	8.7
21708	Gwen	f	14	1994	lac	1011	NA	NA	NA	13-Jul-10	22-Jun-10	-25.5	8.6
21708	Gwen	f	14	1994	lac	997	NA	NA	NA	13-Jul-10	8-Jun-10	-25.6	8.6
21708	Gwen	f	14	1994	lac	983	NA	NA	NA	13-Jul-10	25-May-10	-25.7	8.6
21708	Gwen	f	14	1994	lac	969	NA	NA	NA	13-Jul-10	11-May-10	-25.7	8.5
21708	Gwen	f	14	1994	lac	955	NA	NA	NA	13-Jul-10	27-Apr-10	-25.5	8.5
21708	Gwen	f	14	1994	lac	934	NA	NA	NA	13-Jul-10	6-Apr-10	-25.9	8.8
13506	Gwen	f	14	1994	lac	578	NA	NA	NA	29-Apr-09	15-Apr-09	-25.5	8.2
13506	Gwen	f	14	1994	lac	536	NA	NA	NA	29-Apr-09	4-Mar-09	-25.8	8.4
13507	Gwen	f	14	1994	lac	599	NA	NA	NA	3-Jun-09	6-May-09	-25.5	8.4
13507	Gwen	f	14	1994	lac	529	NA	NA	NA	3-Jun-09	25-Feb-09	-26.0	8.0
21569	Hanna	f	1	1984	lac	1491	15	4	2010	21-Feb-10	31-Jan-10	-25.4	8.6
21569	Hanna	f	1	1984	lac	1477	15	4	2010	21-Feb-10	17-Jan-10	-25.3	8.6
21569	Hanna	f	1	1984	lac	1463	15	4	2010	21-Feb-10	3-Jan-10	-25.4	8.5
21569	Hanna	f	1	1984	lac	1449	15	4	2010	21-Feb-10	20-Dec-09	-25.4	8.4
21569	Hanna	f	1	1984	lac	1435	15	4	2010	21-Feb-10	6-Dec-09	-25.4	8.4
21569	Hanna	f	1	1984	lac	1407	15	4	2010	21-Feb-10	8-Nov-09	-25.3	8.4
21569	Hanna	f	1	1984	lac	1379	15	4	2010	21-Feb-10	11-Oct-09	-25.3	8.3
21569	Hanna	f	1	1984	lac	1365	15	4	2010	21-Feb-10	27-Sep-09	-25.3	8.2
21569	Hanna	f	1	1984	lac	1351	15	4	2010	21-Feb-10	13-Sep-09	-25.2	8.2
21570	Hanna	f	1	1984	lac	1331	15	4	2010	22-Feb-10	24-Aug-09	-25.0	8.0
21604	Hanna	f	1	1984	lac	1527	15	4	2010	22-Mar-10	8-Mar-10	-25.7	8.4
21604	Hanna	f	1	1984	lac	1499	15	4	2010	22-Mar-10	8-Feb-10	-25.8	8.5
21604	Hanna	f	1	1984	lac	1471	15	4	2010	22-Mar-10	11-Jan-10	-25.6	8.5
21613	Hanna	f	1	1984	lac	1536	15	4	2010	31-Mar-10	17-Mar-10	-25.3	8.5
21613	Hanna	f	1	1984	lac	1508	15	4	2010	31-Mar-10	17-Feb-10	-25.4	8.7
21613	Hanna	f	1	1984	lac	1480	15	4	2010	31-Mar-10	20-Jan-10	-25.7	8.5
13508	Hanna	f	1	1984	lac	1171	15	4	2010	14-Apr-09	17-Mar-09	-25.3	8.6
13508	Hanna	f	1	1984	lac	1101	15	4	2010	14-Apr-09	6-Jan-09	-26.0	8.7
13509	Hanna	f	1	1984	lac	1218	15	4	2010	31-May-09	3-May-09	-25.6	8.3
13510	Hanna	f	1	1984	lac	1223	15	4	2010	5-Jun-09	8-May-09	-25.5	8.4
21578	Iris	f	5	1984	preg	-26	NA	NA	NA	3-Mar-10	17-Feb-10	-25.7	8.6
21578	Iris	f	5	1984	preg	-54	NA	NA	NA	3-Mar-10	20-Jan-10	-25.5	8.6
21578	Iris	f	5	1984	preg	-82	NA	NA	NA	3-Mar-10	23-Dec-09	-25.5	8.8
21578	Iris	f	5	1984	preg	-124	NA	NA	NA	3-Mar-10	11-Nov-09	-25.6	8.8
21624	Iris	f	5	1984	lac	6	NA	NA	NA	2-May-10	21-Mar-10	-25.7	8.4
21624	Iris	f	5	1984	preg	-21	NA	NA	NA	3-May-10	22-Feb-10	-25.7	8.4
21624	Iris	f	5	1984	preg	-55	NA	NA	NA	4-May-10	19-Jan-10	-25.5	8.2
21635	Iris	f	5	1984	lac	40	NA	NA	NA	15-May-10	24-Apr-10	-25.7	8.8
21635	Iris	f	5	1984	lac	26	NA	NA	NA	15-May-10	10-Apr-10	-25.7	8.7
21635	Iris	f	5	1984	lac	12	NA	NA	NA	15-May-10	27-Mar-10	-25.8	8.8
21635	Iris	f	5	1984	preg	-2	NA	NA	NA	15-May-10	13-Mar-10	-25.7	8.7
21635	Iris	f	5	1984	preg	-23	NA	NA	NA	15-May-10	20-Feb-10	-25.9	8.7
21699	Iris	f	5	1984	lac	92	NA	NA	NA	29-Jun-10	15-Jun-10	-25.3	8.6
21699	Iris	f	5	1984	lac	64	NA	NA	NA	29-Jun-10	18-May-10	-25.2	8.7
21699	Iris	f	5	1984	lac	36	NA	NA	NA	29-Jun-10	20-Apr-10	-25.6	8.8
21699	Iris	f	5	1984	lac	8	NA	NA	NA	29-Jun-10	23-Mar-10	-26.0	9.1
21699	Iris	f	5	1984	preg	-20	NA	NA	NA	29-Jun-10	23-Feb-10	-26.4	9.0
21713	Iris	f	5	1984	lac	100	NA	NA	NA	14-Jul-10	23-Jun-10	-25.5	8.7
21713	Iris	f	5	1984	lac	58	NA	NA	NA	14-Jul-10	12-May-10	-26.0	9.0
21713	Iris	f	5	1984	lac	9	NA	NA	NA	14-Jul-10	24-Mar-10	-25.6	8.6
21718	Iris	f	5	1984	lac	120	NA	NA	NA	20-Jul-10	13-Jul-10	-25.8	8.9
21718	Iris	f	5	1984	lac	106	NA	NA	NA	20-Jul-10	29-Jun-10	-25.4	8.8
21718	Iris	f	5	1984	lac	92	NA	NA	NA	20-Jul-10	15-Jun-10	-25.5	8.8
21718	Iris	f	5	1984	lac	78	NA	NA	NA	20-Jul-10	1-Jun-10	-25.2	8.6
21718	Iris	f	5	1984	lac	64	NA	NA	NA	20-Jul-10	18-May-10	-25.0	8.6
21718	Iris	f	5	1984	lac	50	NA	NA	NA	20-Jul-10	4-May-10	-24.9	8.8
21718	Iris	f	5	1984	lac	15	NA	NA	NA	20-Jul-10	30-Mar-10	-25.6	8.9
21612	Iris	f	5	1984	lac	2	NA	NA	NA	31-Mar-10	17-Mar-10	-25.4	8.8
21612	Iris	f	5	1984	preg	-26	NA	NA	NA	31-Mar-10	17-Feb-10	-25.5	8.8
21612	Iris	f	5	1984	preg	-54	NA	NA	NA	31-Mar-10	20-Jan-10	-25.6	8.7
21612	Iris	f	5	1984	preg	-82	NA	NA	NA	31-Mar-10	23-Dec-09	-25.4	8.6
12577	Iris	f	5	1984	cyc	-395	NA	NA	NA	13-Mar-09	13-Feb-09	-25.9	8.6
12577	Iris	f	5	1984	cyc	-451	NA	NA	NA	13-Mar-09	19-Dec-08	-25.7	8.5
21587	Luna	f	16	1999	cyc	na	NA	NA	NA	8-Mar-10	22-Feb-10	-25.7	8.5
21587	Luna	f	16	1999	cyc	na	NA	NA	NA	8-Mar-10	25-Jan-10	-26.1	8.9
21587	Luna	f	16	1999	cyc	na	NA	NA	NA	8-Mar-10	28-Dec-09	-26.3	8.6
21630	Luna	f	16	1999	cyc	na	NA	NA	NA	8-May-10	24-Apr-10	-25.9	8.9
21630	Luna	f	16	1999	cyc	na	NA	NA	NA	8-May-10	27-Mar-10	-25.8	9.0
21630	Luna	f	16	1999	cyc	na	NA	NA	NA	8-May-10	27-Feb-10	-26.0	8.9
21630	Luna	f	16	1999	cyc	na	NA	NA	NA	8-May-10	30-Jan-10	-25.9	8.6
21630	Luna	f	16	1999	cyc	na	NA	NA	NA	8-May-10	5-Dec-09	-25.5	8.6
21641	Luna	f	16	1999	cyc	na	NA	NA	NA	17-May-10	5-Apr-10	-25.8	8.6
21641	Luna	f	16	1999	cyc	na	NA	NA	NA	17-May-10	8-Mar-10	-26.0	8.7
21641	Luna	f	16	1999	cyc	na	NA	NA	NA	17-May-10	11-Jan-10	-26.2	8.8
21625	Martha	f	3	1977	cyc	na	NA	NA	NA	4-May-10	20-Apr-10	-25.7	8.7
21625	Martha	f	3	1977	cyc	na	NA	NA	NA	4-May-10	23-Mar-10	-25.7	8.8
21625	Martha	f	3	1977	cyc	na	NA	NA	NA	4-May-10	23-Feb-10	-25.7	8.7
21625	Martha	f	3	1977	cyc	na	NA	NA	NA	4-May-10	12-Jan-10	-25.8	8.6
21632	Martha	f	3	1977	cyc	na	NA	NA	NA	13-May-10	29-Apr-10	-25.9	8.9
21632	Martha	f	3	1977	cyc	na	NA	NA	NA	13-May-10	1-Apr-10	-25.7	8.8
21632	Martha	f	3	1977	cyc	na	NA	NA	NA	13-May-10	4-Mar-10	-25.9	8.7
21632	Martha	f	3	1977	cyc	na	NA	NA	NA	13-May-10	21-Jan-10	-25.8	8.5
21649	Martha	f	3	1977	cyc	na	NA	NA	NA	22-May-10	15-May-10	-25.9	8.7
21649	Martha	f	3	1977	cyc	na	NA	NA	NA	22-May-10	1-May-10	-25.7	8.7
21649	Martha	f	3	1977	cyc	na	NA	NA	NA	22-May-10	17-Apr-10	-25.8	8.9
21649	Martha	f	3	1977	cyc	na	NA	NA	NA	22-May-10	3-Apr-10	-26.0	8.8
21649	Martha	f	3	1977	cyc	na	NA	NA	NA	22-May-10	20-Mar-10	-26.0	8.7
21649	Martha	f	3	1977	cyc	na	NA	NA	NA	22-May-10	6-Mar-10	-26.0	8.5
21649	Martha	f	3	1977	cyc	na	NA	NA	NA	22-May-10	20-Feb-10	-26.0	8.5
21649	Martha	f	3	1977	cyc	na	NA	NA	NA	22-May-10	6-Feb-10	-26.0	8.5
21661	Martha	f	3	1977	cyc	na	NA	NA	NA	4-Jun-10	14-May-10	-25.7	8.8
21661	Martha	f	3	1977	cyc	na	NA	NA	NA	4-Jun-10	30-Apr-10	-25.6	8.8
21661	Martha	f	3	1977	cyc	na	NA	NA	NA	4-Jun-10	16-Apr-10	-25.5	8.6
21661	Martha	f	3	1977	cyc	na	NA	NA	NA	4-Jun-10	2-Apr-10	-25.6	8.7
21661	Martha	f	3	1977	cyc	na	NA	NA	NA	4-Jun-10	19-Mar-10	-25.9	8.8
21661	Martha	f	3	1977	cyc	na	NA	NA	NA	4-Jun-10	5-Mar-10	-26.0	8.8
21661	Martha	f	3	1977	cyc	na	NA	NA	NA	4-Jun-10	12-Feb-10	-26.1	8.7
21661	Martha	f	3	1977	cyc	na	NA	NA	NA	4-Jun-10	15-Jan-10	-26.2	8.6
13513	Martha	f	3	1977	cyc	na	NA	NA	NA	26-May-09	5-May-09	-25.7	8.5
13513	Martha	f	3	1977	cyc	na	NA	NA	NA	26-May-09	14-Apr-09	-26.0	8.8
21573	Olga	f	13	1984	lac	280	NA	NA	NA	26-Feb-10	19-Feb-10	-26.2	8.2
21573	Olga	f	13	1984	lac	266	NA	NA	NA	26-Feb-10	5-Feb-10	-25.4	9.1
21573	Olga	f	13	1984	lac	252	NA	NA	NA	26-Feb-10	22-Jan-10	-25.6	9.1
21573	Olga	f	13	1984	lac	238	NA	NA	NA	26-Feb-10	8-Jan-10	-25.6	9.0
21573	Olga	f	13	1984	lac	224	NA	NA	NA	26-Feb-10	25-Dec-09	-25.6	8.9
21573	Olga	f	13	1984	lac	210	NA	NA	NA	26-Feb-10	11-Dec-09	-25.7	9.0
21573	Olga	f	13	1984	lac	196	NA	NA	NA	26-Feb-10	27-Nov-09	-25.7	8.8
21573	Olga	f	13	1984	lac	182	NA	NA	NA	26-Feb-10	13-Nov-09	-25.8	9.0

21573	Olga	f	13	1984	lac	154	NA	NA	NA	26-Feb-10	16-Oct-09	-26.0	9.0
21628	Olga	f	13	1984	lac	315	NA	NA	NA	7-May-10	26-Mar-10	-25.7	9.2
21628	Olga	f	13	1984	lac	287	NA	NA	NA	7-May-10	26-Feb-10	-25.7	9.3
21714	Olga	f	13	1984	lac	413	NA	NA	NA	16-Jul-10	2-Jul-10	-25.7	9.2
21714	Olga	f	13	1984	lac	385	NA	NA	NA	16-Jul-10	4-Jun-10	-26.0	9.0
21714	Olga	f	13	1984	lac	357	NA	NA	NA	16-Jul-10	7-May-10	-25.6	9.1
13516	Olga	f	13	1984	preg	-13	NA	NA	NA	30-May-09	2-May-09	-25.7	8.8
13516	Olga	f	13	1984	preg	-76	NA	NA	NA	30-May-09	28-Feb-09	-25.6	8.8
13517	Olga	f	13	1984	lac	19	NA	NA	NA	17-Jun-09	3-Jun-09	-25.7	8.6
13517	Olga	f	13	1984	preg	-9	NA	NA	NA	17-Jun-09	6-May-09	-25.8	8.7
13517	Olga	f	13	1984	preg	-72	NA	NA	NA	17-Jun-09	4-Mar-09	-25.5	8.6
13518	Olga	f	13	1984	lac	21	NA	NA	NA	19-Jun-09	5-Jun-09	-25.2	8.7
13518	Olga	f	13	1984	preg	-7	NA	NA	NA	19-Jun-09	8-May-09	-25.6	8.5
13518	Olga	f	13	1984	preg	-35	NA	NA	NA	19-Jun-09	10-Apr-09	-25.9	8.7
13518	Olga	f	13	1984	preg	-63	NA	NA	NA	19-Jun-09	13-Mar-09	-25.8	8.2
14099	Olga	f	13	1984	lac	53	NA	NA	NA	28-Jul-09	7-Jul-09	-25.7	8.9
14099	Olga	f	13	1984	lac	11	NA	NA	NA	28-Jul-09	26-May-09	-26.1	8.7
14099	Olga	f	13	1984	preg	-31	NA	NA	NA	28-Jul-09	14-Apr-09	-26.1	8.4
21590	Paula	f	6	1984	preg	-111	NA	NA	NA	10-Mar-10	24-Feb-10	-25.9	8.8
21590	Paula	f	6	1984	preg	-139	NA	NA	NA	10-Mar-10	27-Jan-10	-26.0	8.8
21590	Paula	f	6	1984	preg	-167	NA	NA	NA	10-Mar-10	30-Dec-09	-25.8	8.5
21590	Paula	f	6	1984	preg	-209	NA	NA	NA	10-Mar-10	18-Nov-09	-25.7	8.4
21605	Paula	f	6	1984	preg	-104	NA	NA	NA	24-Mar-10	3-Mar-10	-26.1	8.9
21605	Paula	f	6	1984	preg	-118	NA	NA	NA	24-Mar-10	17-Feb-10	-26.3	9.0
21605	Paula	f	6	1984	preg	-132	NA	NA	NA	24-Mar-10	3-Feb-10	-26.3	8.9
21605	Paula	f	6	1984	preg	-146	NA	NA	NA	24-Mar-10	20-Jan-10	-26.4	8.9
21605	Paula	f	6	1984	preg	-160	NA	NA	NA	24-Mar-10	6-Jan-10	-26.3	8.8
21605	Paula	f	6	1984	preg	-174	NA	NA	NA	24-Mar-10	23-Dec-09	-26.3	8.7
21605	Paula	f	6	1984	preg	-195	NA	NA	NA	24-Mar-10	2-Dec-09	-26.3	8.5
21634	Paula	f	6	1984	preg	-53	NA	NA	NA	14-May-10	23-Apr-10	-25.9	9.1
21634	Paula	f	6	1984	preg	-95	NA	NA	NA	14-May-10	12-Mar-10	-26.1	9.1
21634	Paula	f	6	1984	preg	-144	NA	NA	NA	14-May-10	22-Jan-10	-25.8	8.9
21653	Paula	f	6	1984	preg	-28	NA	NA	NA	25-May-10	18-May-10	-25.8	8.7
21653	Paula	f	6	1984	preg	-42	NA	NA	NA	25-May-10	4-May-10	-25.8	8.7
21653	Paula	f	6	1984	preg	-56	NA	NA	NA	25-May-10	20-Apr-10	-25.9	8.7
21653	Paula	f	6	1984	preg	-70	NA	NA	NA	25-May-10	6-Apr-10	-25.6	8.8
21653	Paula	f	6	1984	preg	-84	NA	NA	NA	25-May-10	23-Mar-10	-25.7	8.9
21653	Paula	f	6	1984	preg	-98	NA	NA	NA	25-May-10	9-Mar-10	-26.0	8.9
21653	Paula	f	6	1984	preg	-112	NA	NA	NA	25-May-10	23-Feb-10	-26.0	8.9
21653	Paula	f	6	1984	preg	-126	NA	NA	NA	25-May-10	9-Feb-10	-26.2	8.9
21653	Paula	f	6	1984	preg	-140	NA	NA	NA	25-May-10	26-Jan-10	-26.1	9.0
21653	Paula	f	6	1984	preg	-168	NA	NA	NA	25-May-10	29-Dec-09	-26.2	8.5
21687	Paula	f	6	1984	preg	-10	NA	NA	NA	19-Jun-10	5-Jun-10	-25.8	9.1
21687	Paula	f	6	1984	preg	-38	NA	NA	NA	19-Jun-10	8-May-10	-26.2	9.2
21687	Paula	f	6	1984	preg	-87	NA	NA	NA	19-Jun-10	20-Mar-10	-25.7	8.7
21707	Paula	f	6	1984	lac	21	NA	NA	NA	13-Jul-10	6-Jul-10	-25.4	8.5
21707	Paula	f	6	1984	lac	7	NA	NA	NA	13-Jul-10	22-Jun-10	-25.4	8.7
21707	Paula	f	6	1984	preg	-7	NA	NA	NA	13-Jul-10	8-Jun-10	-25.3	8.6
21707	Paula	f	6	1984	preg	-21	NA	NA	NA	13-Jul-10	25-May-10	-25.6	8.7
21707	Paula	f	6	1984	preg	-35	NA	NA	NA	13-Jul-10	11-May-10	-25.7	8.8
21707	Paula	f	6	1984	preg	-49	NA	NA	NA	13-Jul-10	27-Apr-10	-25.8	8.9
21707	Paula	f	6	1984	preg	-70	NA	NA	NA	13-Jul-10	6-Apr-10	-26.0	8.9
12580	Paula	f	6	1984	cyc	-489	NA	NA	NA	11-Mar-09	11-Feb-09	-26.0	8.9
12580	Paula	f	6	1984	cyc	-545	NA	NA	NA	11-Mar-09	17-Dec-08	-25.9	8.8
13520	Paula	f	6	1984	cyc	-423	NA	NA	NA	16-May-09	18-Apr-09	-25.8	8.6
13520	Paula	f	6	1984	cyc	-500	NA	NA	NA	16-May-09	31-Jan-09	-26.0	8.9
13521	Paula	f	6	1984	cyc	-404	NA	NA	NA	4-Jun-09	7-May-09	-25.8	8.3
13521	Paula	f	6	1984	cyc	-460	NA	NA	NA	4-Jun-09	12-Mar-09	-26.0	8.6
13521	Paula	f	6	1984	cyc	-530	NA	NA	NA	4-Jun-09	1-Jan-09	-26.3	8.7
21591	Polly	f	22	2002	cyc	na	NA	NA	NA	12-Mar-10	26-Feb-10	-25.6	8.8
21591	Polly	f	22	2002	cyc	na	NA	NA	NA	12-Mar-10	29-Jan-10	-25.4	8.8
21591	Polly	f	22	2002	cyc	na	NA	NA	NA	12-Mar-10	1-Jan-10	-25.6	8.9
21591	Polly	f	22	2002	cyc	na	NA	NA	NA	12-Mar-10	4-Dec-09	-25.9	8.7
21591	Polly	f	22	2002	cyc	na	NA	NA	NA	12-Mar-10	9-Oct-09	-26.0	8.4
21620	Polly	f	22	2002	cyc	na	NA	NA	NA	15-Apr-10	18-Mar-10	-25.1	8.9
21620	Polly	f	22	2002	cyc	na	NA	NA	NA	15-Apr-10	21-Jan-10	-26.2	9.4
21709	Polly	f	22	2002	cyc	na	NA	NA	NA	13-Jul-10	6-Jul-10	-25.4	9.0
21709	Polly	f	22	2002	cyc	na	NA	NA	NA	13-Jul-10	22-Jun-10	-25.6	9.0
21709	Polly	f	22	2002	cyc	na	NA	NA	NA	13-Jul-10	8-Jun-10	-25.4	9.0
21709	Polly	f	22	2002	cyc	na	NA	NA	NA	13-Jul-10	25-May-10	-25.7	9.1
21709	Polly	f	22	2002	cyc	na	NA	NA	NA	13-Jul-10	11-May-10	-25.7	9.1
21709	Polly	f	22	2002	cyc	na	NA	NA	NA	13-Jul-10	27-Apr-10	-25.8	9.0
21709	Polly	f	22	2002	cyc	na	NA	NA	NA	13-Jul-10	6-Apr-10	-25.4	8.7
21629	Rio	f	2	1984	cyc	1945	NA	NA	NA	7-May-10	30-Apr-10	-25.2	8.8
21629	Rio	f	2	1984	cyc	1931	NA	NA	NA	7-May-10	16-Apr-10	-25.3	8.9
21629	Rio	f	2	1984	cyc	1917	NA	NA	NA	7-May-10	2-Apr-10	-25.5	8.9
21629	Rio	f	2	1984	cyc	1903	NA	NA	NA	7-May-10	19-Mar-10	-25.6	8.8
21629	Rio	f	2	1984	cyc	1889	NA	NA	NA	7-May-10	5-Mar-10	-25.8	8.8
21629	Rio	f	2	1984	cyc	1875	NA	NA	NA	7-May-10	19-Feb-10	-25.9	8.7
21629	Rio	f	2	1984	cyc	1861	NA	NA	NA	7-May-10	5-Feb-10	-26.0	8.8
21629	Rio	f	2	1984	cyc	1847	NA	NA	NA	7-May-10	22-Jan-10	-26.1	8.8
21629	Rio	f	2	1984	cyc	1819	NA	NA	NA	7-May-10	25-Dec-09	-25.8	8.5
21681	Rio	f	2	1984	cyc	1987	NA	NA	NA	18-Jun-10	11-Jun-10	-25.1	8.7
21681	Rio	f	2	1984	cyc	1973	NA	NA	NA	18-Jun-10	28-May-10	-25.2	9.0
21681	Rio	f	2	1984	cyc	1959	NA	NA	NA	18-Jun-10	14-May-10	-25.3	9.0
21681	Rio	f	2	1984	cyc	1945	NA	NA	NA	18-Jun-10	30-Apr-10	-25.5	8.9
21681	Rio	f	2	1984	cyc	1931	NA	NA	NA	18-Jun-10	16-Apr-10	-25.6	9.0
21681	Rio	f	2	1984	cyc	1917	NA	NA	NA	18-Jun-10	2-Apr-10	-25.8	9.1
21681	Rio	f	2	1984	cyc	1903	NA	NA	NA	18-Jun-10	19-Mar-10	-25.7	9.1
21681	Rio	f	2	1984	cyc	1861	NA	NA	NA	18-Jun-10	5-Feb-10	-25.7	8.7
21681	Rio	f	2	1984	cyc	1840	NA	NA	NA	18-Jun-10	15-Jan-10	-26.1	8.5
21695	Rio	f	2	1984	cyc	1988	NA	NA	NA	26-Jun-10	12-Jun-10	-25.2	9.1
21695	Rio	f	2	1984	cyc	1960	NA	NA	NA	26-Jun-10	15-May-10	-26.1	9.0
21695	Rio	f	2	1984	cyc	1925	NA	NA	NA	26-Jun-10	10-Apr-10	-26.0	8.9
12579	Rio	f	2	1984	lac	1522	NA	NA	NA	31-Mar-09	3-Mar-09	-25.7	8.6
12579	Rio	f	2	1984	lac	1466	NA	NA	NA	31-Mar-09	6-Jan-09	-25.9	8.8
13522	Rio	f	2	1984	lac	1590	NA	NA	NA	7-Jun-09	10-May-09	-25.3	8.4
13522	Rio	f	2	1984	lac	1520	NA	NA	NA	7-Jun-09	1-Mar-09	-25.2	8.5
21654	Susi	f	15	1994	lac	354	NA	NA	NA	25-May-10	4-May-10	-25.3	9.0
21654	Susi	f	15	1994	lac	340	NA	NA	NA	25-May-10	20-Apr-10	-25.4	9.1
21654	Susi	f	15	1994	lac	326	NA	NA	NA	25-May-10	6-Apr-10	-25.7	8.9
21654	Susi	f	15	1994	lac	312	NA	NA	NA	25-May-10	23-Mar-10	-25.8	9.1
21654	Susi	f	15	1994	lac	298	NA	NA	NA	25-May-10	9-Mar-10	-25.8	9.0
21654	Susi	f	15	1994	lac	284	NA	NA	NA	25-May-10	23-Feb-10	-25.7	8.9
21654	Susi	f	15	1994	lac	256	NA	NA	NA	25-May-10	26-Jan-10	-25.5	9.0
21696	Susi	f	15	1994	lac	387	NA	NA	NA	27-Jun-10	6-Jun-10	-24.8	9.0
21696	Susi	f	15	1994	lac	345	NA	NA	NA	27-Jun-10	25-Apr-10	-25.6	9.1
21627	Uma	f	20	1994	lac	623	NA	NA	NA	7-May-10	30-Apr-10	-24.7	8.7
21627	Uma	f	20	1994	lac	609	NA	NA	NA	7-May-10	16-Apr-10	-24.9	8.9
21627	Uma	f	20	1994	lac	595	NA	NA	NA	7-May-10	2-Apr-10	-25.1	8.6
21627	Uma	f	20	1994	lac	581	NA	NA	NA	7-May-10	19-Mar-10	-25.3	8.6
21627	Uma	f	20	1994	lac	567	NA	NA	NA	7-May-10	5-Mar-10	-25.5	8.6
21627	Uma	f	20	1994	lac	553	NA	NA	NA	7-May-10	19-Feb-10	-25.7	8.4
21627	Uma	f	20	1994	lac	525	NA	NA	NA	7-May-10	22-Jan-10	-25.7	8.2
21702	Uma	f	20	1994	lac	662	NA	NA	NA	29-Jun-10	8-Jun-10	-24.9	8.0
21702	Uma	f	20	1994	lac	648	NA	NA	NA	29-Jun-10	25-May-10	-24.8	8.4

21702	Uma	f	20	1994	lac	634	NA	NA	NA	29-Jun-10	11-May-10	-25.1	8.7
21702	Uma	f	20	1994	lac	620	NA	NA	NA	29-Jun-10	27-Apr-10	-25.1	8.7
21702	Uma	f	20	1994	lac	606	NA	NA	NA	29-Jun-10	13-Apr-10	-25.5	8.7
21702	Uma	f	20	1994	lac	592	NA	NA	NA	29-Jun-10	30-Mar-10	-25.6	8.7
21702	Uma	f	20	1994	lac	564	NA	NA	NA	29-Jun-10	2-Mar-10	-25.8	8.5
21717	Uma	f	20	1994	lac	697	NA	NA	NA	20-Jul-10	13-Jul-10	-25.4	8.2
21717	Uma	f	20	1994	lac	683	NA	NA	NA	20-Jul-10	29-Jun-10	-25.2	8.2
21717	Uma	f	20	1994	lac	669	NA	NA	NA	20-Jul-10	15-Jun-10	-25.2	8.4
21717	Uma	f	20	1994	lac	655	NA	NA	NA	20-Jul-10	1-Jun-10	-25.1	8.5
21717	Uma	f	20	1994	lac	641	NA	NA	NA	20-Jul-10	18-May-10	-25.2	8.5
21717	Uma	f	20	1994	lac	627	NA	NA	NA	20-Jul-10	4-May-10	-25.2	8.6
21717	Uma	f	20	1994	lac	606	NA	NA	NA	20-Jul-10	13-Apr-10	-25.2	8.2
21637	Uma	f	20	1994	lac	625	NA	NA	NA	16-May-10	2-May-10	-25.4	8.5
21637	Uma	f	20	1994	lac	597	NA	NA	NA	16-May-10	4-Apr-10	-25.5	8.6
21637	Uma	f	20	1994	lac	569	NA	NA	NA	16-May-10	7-Mar-10	-26.1	8.6
21683	Uma	f	20	1994	lac	666	NA	NA	NA	19-Jun-10	12-Jun-10	-24.9	8.1
21683	Uma	f	20	1994	lac	652	NA	NA	NA	19-Jun-10	29-May-10	-24.7	8.2
21683	Uma	f	20	1994	lac	638	NA	NA	NA	19-Jun-10	15-May-10	-24.8	8.5
21683	Uma	f	20	1994	lac	624	NA	NA	NA	19-Jun-10	1-May-10	-25.0	8.7
21683	Uma	f	20	1994	lac	610	NA	NA	NA	19-Jun-10	17-Apr-10	-25.2	8.7
21683	Uma	f	20	1994	lac	596	NA	NA	NA	19-Jun-10	3-Apr-10	-25.4	8.7
21683	Uma	f	20	1994	lac	582	NA	NA	NA	19-Jun-10	20-Mar-10	-25.5	8.7
21683	Uma	f	20	1994	lac	568	NA	NA	NA	19-Jun-10	6-Mar-10	-25.6	8.5
21683	Uma	f	20	1994	lac	547	NA	NA	NA	19-Jun-10	13-Feb-10	-25.8	8.4
12578	Uma	f	20	1994	lac	187	NA	NA	NA	18-Mar-09	18-Feb-09	-25.9	8.6
13525	Venus	f	na	1999	na	na	NA	NA	NA	21-Jun-09	24-May-09	-25.4	8.3
13525	Venus	f	na	1999	na	na	NA	NA	NA	21-Jun-09	15-Mar-09	-25.6	8.2
21677	Wilma	f	21	1999	cyc	-267	NA	NA	NA	14-Jun-10	24-May-10	-25.0	8.9
21677	Wilma	f	21	1999	cyc	-295	NA	NA	NA	14-Jun-10	26-Apr-10	-24.8	8.8
21677	Wilma	f	21	1999	cyc	-309	NA	NA	NA	14-Jun-10	12-Apr-10	-24.8	8.9
21677	Wilma	f	21	1999	cyc	-323	NA	NA	NA	14-Jun-10	29-Mar-10	-25.1	8.9
21677	Wilma	f	21	1999	cyc	-337	NA	NA	NA	14-Jun-10	15-Mar-10	-25.3	9.1
21677	Wilma	f	21	1999	cyc	-351	NA	NA	NA	14-Jun-10	1-Mar-10	-25.7	9.1
21677	Wilma	f	21	1999	cyc	-365	NA	NA	NA	14-Jun-10	15-Feb-10	-25.9	9.3
21677	Wilma	f	21	1999	cyc	-379	NA	NA	NA	14-Jun-10	1-Feb-10	-26.1	9.1
21710	Wilma	f	21	1999	cyc	-237	NA	NA	NA	14-Jul-10	23-Jun-10	-25.6	9.6
21710	Wilma	f	21	1999	cyc	-251	NA	NA	NA	14-Jul-10	9-Jun-10	-25.3	8.9
21710	Wilma	f	21	1999	cyc	-265	NA	NA	NA	14-Jul-10	26-May-10	-25.2	9.0
21710	Wilma	f	21	1999	cyc	-279	NA	NA	NA	14-Jul-10	12-May-10	-25.2	8.9
21710	Wilma	f	21	1999	cyc	-293	NA	NA	NA	14-Jul-10	28-Apr-10	-25.2	9.1
21710	Wilma	f	21	1999	cyc	-307	NA	NA	NA	14-Jul-10	14-Apr-10	-25.3	9.2
21710	Wilma	f	21	1999	cyc	-321	NA	NA	NA	14-Jul-10	31-Mar-10	-25.3	9.2
21600	Zoe	f	9	1984	preg	-105	NA	NA	NA	16-Mar-10	2-Mar-10	-25.8	8.8
21600	Zoe	f	9	1984	preg	-133	NA	NA	NA	16-Mar-10	2-Feb-10	-25.7	8.7
21600	Zoe	f	9	1984	preg	-154	NA	NA	NA	16-Mar-10	12-Jan-10	-25.7	8.7
21600	Zoe	f	9	1984	preg	-168	NA	NA	NA	16-Mar-10	29-Dec-09	-25.8	8.8
21600	Zoe	f	9	1984	preg	-182	NA	NA	NA	16-Mar-10	15-Dec-09	-25.9	8.5
21600	Zoe	f	9	1984	preg	-210	NA	NA	NA	16-Mar-10	17-Nov-09	-25.8	8.5
21645	Zoe	f	9	1984	preg	-47	NA	NA	NA	20-May-10	29-Apr-10	-25.3	9.2
21645	Zoe	f	9	1984	preg	-61	NA	NA	NA	20-May-10	15-Apr-10	-25.3	9.3
21645	Zoe	f	9	1984	preg	-75	NA	NA	NA	20-May-10	1-Apr-10	-25.5	9.3
21645	Zoe	f	9	1984	preg	-89	NA	NA	NA	20-May-10	18-Mar-10	-25.8	9.3
21645	Zoe	f	9	1984	preg	-103	NA	NA	NA	20-May-10	4-Mar-10	-25.9	9.2
21645	Zoe	f	9	1984	preg	-138	NA	NA	NA	20-May-10	28-Jan-10	-26.0	9.2
21722	Zoe	f	9	1984	lac	22	NA	NA	NA	21-Jul-10	7-Jul-10	-25.3	8.6
21722	Zoe	f	9	1984	preg	-6	NA	NA	NA	21-Jul-10	9-Jun-10	-25.5	8.6
21722	Zoe	f	9	1984	preg	-34	NA	NA	NA	21-Jul-10	12-May-10	-25.4	8.5
21722	Zoe	f	9	1984	preg	-76	NA	NA	NA	21-Jul-10	31-Mar-10	-26.0	8.7
21722	Zoe	f	9	1984	preg	-132	NA	NA	NA	21-Jul-10	3-Feb-10	-26.5	8.5
13527	Zoe	f	9	1984	cyc	-392	NA	NA	NA	16-Jun-09	19-May-09	-25.5	8.1
13527	Zoe	f	9	1984	cyc	-469	NA	NA	NA	16-Jun-09	3-Mar-09	-25.7	8.4
14096	Zoe	f	9	1984	cyc	-381	NA	NA	NA	4-Jul-09	30-May-09	-25.9	8.2
14096	Zoe	f	9	1984	cyc	-465	NA	NA	NA	4-Jul-09	7-Mar-09	-25.8	8.3
12573	Apollo	m	17	1999	na	na	15	3	2010	6-Mar-09	20-Feb-09	-25.7	7.8
12573	Apollo	m	17	1999	na	na	15	3	2010	6-Mar-09	23-Jan-09	-25.8	8.0
12573	Apollo	m	17	1999	na	na	15	3	2010	6-Mar-09	28-Nov-08	-25.8	8.0
13529	Apollo	m	17	1999	na	na	15	3	2010	29-May-09	15-May-09	-25.6	8.1
21598	Ben	m	12	1994	na	na	NA	NA	NA	14-Mar-10	28-Feb-10	-25.7	8.9
21598	Ben	m	12	1994	na	na	NA	NA	NA	14-Mar-10	31-Jan-10	-25.9	8.9
21598	Ben	m	12	1994	na	na	NA	NA	NA	14-Mar-10	3-Jan-10	-25.8	8.8
21598	Ben	m	12	1994	na	na	NA	NA	NA	14-Mar-10	6-Dec-09	-25.6	8.6
21598	Ben	m	12	1994	na	na	NA	NA	NA	14-Mar-10	1-Nov-09	-25.6	8.5
21603	Ben	m	12	1994	na	na	NA	NA	NA	20-Mar-10	6-Mar-10	-25.7	8.9
21603	Ben	m	12	1994	na	na	NA	NA	NA	20-Mar-10	6-Feb-10	-25.7	8.9
21603	Ben	m	12	1994	na	na	NA	NA	NA	20-Mar-10	9-Jan-10	-25.6	8.6
21607	Ben	m	12	1994	na	na	NA	NA	NA	26-Mar-10	5-Mar-10	-25.8	8.9
21607	Ben	m	12	1994	na	na	NA	NA	NA	26-Mar-10	19-Feb-10	-25.7	8.9
21607	Ben	m	12	1994	na	na	NA	NA	NA	26-Mar-10	5-Feb-10	-25.7	8.8
21607	Ben	m	12	1994	na	na	NA	NA	NA	26-Mar-10	22-Jan-10	-25.7	8.8
21607	Ben	m	12	1994	na	na	NA	NA	NA	26-Mar-10	8-Jan-10	-25.9	8.8
21607	Ben	m	12	1994	na	na	NA	NA	NA	26-Mar-10	25-Dec-09	-25.7	8.7
21607	Ben	m	12	1994	na	na	NA	NA	NA	26-Mar-10	11-Dec-09	-25.9	8.6
21607	Ben	m	12	1994	na	na	NA	NA	NA	26-Mar-10	20-Nov-09	-25.7	8.5
21618	Ben	m	12	1994	na	na	NA	NA	NA	7-Apr-10	31-Mar-10	-25.9	9.0
21618	Ben	m	12	1994	na	na	NA	NA	NA	7-Apr-10	17-Mar-10	-25.6	8.9
21618	Ben	m	12	1994	na	na	NA	NA	NA	7-Apr-10	3-Mar-10	-25.7	8.9
21618	Ben	m	12	1994	na	na	NA	NA	NA	7-Apr-10	17-Feb-10	-25.6	8.8
21618	Ben	m	12	1994	na	na	NA	NA	NA	7-Apr-10	3-Feb-10	-26.3	8.8
21618	Ben	m	12	1994	na	na	NA	NA	NA	7-Apr-10	20-Jan-10	-26.1	8.7
21618	Ben	m	12	1994	na	na	NA	NA	NA	7-Apr-10	6-Jan-10	-25.9	8.6
21618	Ben	m	12	1994	na	na	NA	NA	NA	7-Apr-10	23-Dec-09	-25.6	8.3
21618	Ben	m	12	1994	na	na	NA	NA	NA	7-Apr-10	9-Dec-09	-25.6	8.1
21638	Ben	m	12	1994	na	na	NA	NA	NA	16-May-10	2-May-10	-25.9	9.0
21638	Ben	m	12	1994	na	na	NA	NA	NA	16-May-10	4-Apr-10	-26.2	8.9
21638	Ben	m	12	1994	na	na	NA	NA	NA	16-May-10	7-Mar-10	-26.1	8.8
21638	Ben	m	12	1994	na	na	NA	NA	NA	16-May-10	7-Feb-10	-26.1	8.8
21638	Ben	m	12	1994	na	na	NA	NA	NA	16-May-10	10-Jan-10	-25.8	8.6
21703	Ben	m	12	1994	na	na	NA	NA	NA	26-Jun-10	19-Jun-10	-25.3	8.9
21703	Ben	m	12	1994	na	na	NA	NA	NA	27-Jun-10	6-Jun-10	-25.5	9.0
21703	Ben	m	12	1994	na	na	NA	NA	NA	28-Jun-10	24-May-10	-25.6	8.9
21703	Ben	m	12	1994	na	na	NA	NA	NA	29-Jun-10	11-May-10	-25.8	8.9
21703	Ben	m	12	1994	na	na	NA	NA	NA	30-Jun-10	28-Apr-10	-25.7	8.8
21703	Ben	m	12	1994	na	na	NA	NA	NA	1-Jul-10	15-Apr-10	-25.8	8.8
21703	Ben	m	12	1994	na	na	NA	NA	NA	2-Jul-10	26-Mar-10	-25.9	8.8
13530	Ben	m	12	1994	na	na	NA	NA	NA	1-May-09	3-Apr-09	-25.6	8.5
13530	Ben	m	12	1994	na	na	NA	NA	NA	1-May-09	23-Jan-09	-25.9	8.5
13531	Ben	m	12	1994	na	na	NA	NA	NA	10-Jun-09	13-May-09	-25.7	8.3
13531	Ben	m	12	1994	na	na	NA	NA	NA	10-Jun-09	18-Mar-09	-25.4	8.4
13532	Cammillo	m	7	1992	na	na	NA	NA	NA	5-Apr-09	22-Mar-09	-25.9	8.8
13532	Cammillo	m	7	1992	na	na	NA	NA	NA	5-Apr-09	22-Feb-09	-25.9	8.8
13532	Cammillo	m	7	1992	na	na	NA	NA	NA	5-Apr-09	25-Jan-09	-26.2	9.0
13532	Cammillo	m	7	1992	na	na	NA	NA	NA	5-Apr-09	14-Dec-08	-26.2	9.0
14101	Cammillo	m	7	1992	na	na	NA	NA	NA	15-Jul-09	24-Jun-09	-25.5	8.4
14101	Cammillo	m	7	1992	na	na	NA	NA	NA	15-Jul-09	13-May-09	-25.7	8.4

14101	Cammillo	m	7	1992	na	na	NA	NA	NA	15-Jul-09	11-Mar-09	-25.7	8.3
21644	Cammillo	m	7	1992	na	na	NA	NA	NA	19-May-10	12-May-10	-25.0	8.6
21644	Cammillo	m	7	1992	na	na	NA	NA	NA	19-May-10	28-Apr-10	-24.9	8.8
21644	Cammillo	m	7	1992	na	na	NA	NA	NA	19-May-10	14-Apr-10	-25.3	8.8
21644	Cammillo	m	7	1992	na	na	NA	NA	NA	19-May-10	31-Mar-10	-25.5	9.4
21644	Cammillo	m	7	1992	na	na	NA	NA	NA	19-May-10	17-Mar-10	-25.7	8.5
21644	Cammillo	m	7	1992	na	na	NA	NA	NA	19-May-10	3-Mar-10	-25.8	9.0
21644	Cammillo	m	7	1992	na	na	NA	NA	NA	19-May-10	10-Feb-10	-25.9	8.4
21608	Cammillo	m	7	1992	na	na	NA	NA	NA	26-Mar-10	12-Mar-10	-25.4	8.8
21608	Cammillo	m	7	1992	na	na	NA	NA	NA	26-Mar-10	12-Feb-10	-25.7	8.5
21608	Cammillo	m	7	1992	na	na	NA	NA	NA	26-Mar-10	15-Jan-10	-25.7	8.6
21711	Cammillo	m	7	1992	na	na	NA	NA	NA	14-Jul-10	23-Jun-10	-25.5	8.8
21711	Cammillo	m	7	1992	na	na	NA	NA	NA	14-Jul-10	9-Jun-10	-25.5	8.8
21711	Cammillo	m	7	1992	na	na	NA	NA	NA	14-Jul-10	26-May-10	-25.5	8.8
21711	Cammillo	m	7	1992	na	na	NA	NA	NA	14-Jul-10	28-Apr-10	-25.5	9.1
21711	Cammillo	m	7	1992	na	na	NA	NA	NA	14-Jul-10	14-Apr-10	-25.4	8.9
21711	Cammillo	m	7	1992	na	na	NA	NA	NA	14-Jul-10	31-Mar-10	-25.1	8.8
21711	Cammillo	m	7	1992	na	na	NA	NA	NA	14-Jul-10	3-Mar-10	-25.1	8.7
21650	Cammillo	m	7	1992	na	na	NA	NA	NA	22-May-10	8-May-10	-25.6	9.0
21650	Cammillo	m	7	1992	na	na	NA	NA	NA	22-May-10	10-Apr-10	-25.9	9.0
21650	Cammillo	m	7	1992	na	na	NA	NA	NA	22-May-10	13-Mar-10	-25.9	8.8
21650	Cammillo	m	7	1992	na	na	NA	NA	NA	22-May-10	23-Jan-10	-25.9	8.8
21660	Cammillo	m	7	1992	na	na	NA	NA	NA	4-Jun-10	21-May-10	-25.3	9.1
21660	Cammillo	m	7	1992	na	na	NA	NA	NA	4-Jun-10	23-Apr-10	-25.7	8.9
21660	Cammillo	m	7	1992	na	na	NA	NA	NA	4-Jun-10	26-Mar-10	-26.0	9.1
21660	Cammillo	m	7	1992	na	na	NA	NA	NA	4-Jun-10	12-Feb-10	-25.8	8.7
13533	Dante	m	11	1984	na	na	15	7	2010	12-Apr-09	29-Mar-09	-25.8	8.4
13533	Dante	m	11	1984	na	na	15	7	2010	12-Apr-09	1-Mar-09	-26.0	8.6
13533	Dante	m	11	1984	na	na	15	7	2010	12-Apr-09	4-Jan-09	-26.0	9.1
13534	Dante	m	11	1984	na	na	15	7	2010	27-May-09	22-Apr-09	-25.6	8.9
13534	Dante	m	11	1984	na	na	15	7	2010	27-May-09	28-Jan-09	-25.3	8.9
21602	Emil	m	19	1998	na	na	NA	NA	NA	17-Mar-10	3-Mar-10	-25.8	8.6
21602	Emil	m	19	1998	na	na	NA	NA	NA	17-Mar-10	3-Feb-10	-25.7	8.7
21602	Emil	m	19	1998	na	na	NA	NA	NA	17-Mar-10	6-Jan-10	-25.7	8.7
21619	Emil	m	19	1998	na	na	NA	NA	NA	8-Apr-10	18-Mar-10	-26.3	8.6
21619	Emil	m	19	1998	na	na	NA	NA	NA	8-Apr-10	4-Mar-10	-26.2	8.6
21619	Emil	m	19	1998	na	na	NA	NA	NA	8-Apr-10	18-Feb-10	-26.0	8.5
21619	Emil	m	19	1998	na	na	NA	NA	NA	8-Apr-10	4-Feb-10	-25.8	8.7
21619	Emil	m	19	1998	na	na	NA	NA	NA	8-Apr-10	21-Jan-10	-25.8	8.7
21619	Emil	m	19	1998	na	na	NA	NA	NA	8-Apr-10	31-Dec-09	-25.9	8.8
13536	Emil	m	19	1998	na	na	NA	NA	NA	22-Apr-09	18-Mar-09	-25.5	8.4
13536	Emil	m	19	1998	na	na	NA	NA	NA	22-Apr-09	7-Jan-09	-25.8	8.6
13535	Emil	m	19	1998	na	na	NA	NA	NA	5-Apr-09	29-Mar-09	-25.6	8.2
13535	Emil	m	19	1998	na	na	NA	NA	NA	5-Apr-09	15-Mar-09	-25.7	8.1
13535	Emil	m	19	1998	na	na	NA	NA	NA	5-Apr-09	1-Mar-09	-25.8	8.2
13535	Emil	m	19	1998	na	na	NA	NA	NA	5-Apr-09	1-Mar-09	-26.2	8.4
13535	Emil	m	19	1998	na	na	NA	NA	NA	5-Apr-09	1-Feb-09	-26.2	8.5
21616	Jack	m	10	1987	na	na	NA	NA	NA	6-Apr-10	16-Mar-10	-25.8	8.8
21616	Jack	m	10	1987	na	na	NA	NA	NA	6-Apr-10	2-Mar-10	-25.7	8.7
21616	Jack	m	10	1987	na	na	NA	NA	NA	6-Apr-10	16-Feb-10	-25.6	9.3
21616	Jack	m	10	1987	na	na	NA	NA	NA	6-Apr-10	26-Jan-10	-25.9	8.9
21616	Jack	m	10	1987	na	na	NA	NA	NA	6-Apr-10	22-Dec-09	-26.0	8.9
21636	Jack	m	10	1987	na	na	NA	NA	NA	16-May-10	9-May-10	-26.0	8.9
21636	Jack	m	10	1987	na	na	NA	NA	NA	16-May-10	25-Apr-10	-25.9	9.0
21636	Jack	m	10	1987	na	na	NA	NA	NA	16-May-10	11-Apr-10	-25.7	8.9
21636	Jack	m	10	1987	na	na	NA	NA	NA	16-May-10	28-Mar-10	-25.9	8.9
21636	Jack	m	10	1987	na	na	NA	NA	NA	16-May-10	14-Mar-10	-25.9	8.7
21636	Jack	m	10	1987	na	na	NA	NA	NA	16-May-10	28-Feb-10	-25.6	8.8
21636	Jack	m	10	1987	na	na	NA	NA	NA	16-May-10	14-Feb-10	-25.3	8.6
21636	Jack	m	10	1987	na	na	NA	NA	NA	16-May-10	31-Jan-10	-25.2	8.5
21636	Jack	m	10	1987	na	na	NA	NA	NA	16-May-10	17-Jan-10	-25.1	8.7
21636	Jack	m	10	1987	na	na	NA	NA	NA	16-May-10	3-Jan-10	-25.1	8.6
21636	Jack	m	10	1987	na	na	NA	NA	NA	16-May-10	13-Dec-09	-25.3	8.5
21636	Jack	m	10	1987	na	na	NA	NA	NA	16-May-10	15-Nov-09	-25.0	8.5
21665	Jack	m	10	1987	na	na	NA	NA	NA	6-Jun-10	23-May-10	-25.1	8.7
21665	Jack	m	10	1987	na	na	NA	NA	NA	6-Jun-10	25-Apr-10	-24.9	8.6
21665	Jack	m	10	1987	na	na	NA	NA	NA	6-Jun-10	28-Mar-10	-24.8	8.5
21665	Jack	m	10	1987	na	na	NA	NA	NA	6-Jun-10	28-Feb-10	-24.7	8.6
21665	Jack	m	10	1987	na	na	NA	NA	NA	6-Jun-10	17-Jan-10	-24.8	8.6
13537	Jack	m	10	1987	na	na	NA	NA	NA	6-Jun-09	9-May-09	-25.3	8.3
13537	Jack	m	10	1987	na	na	NA	NA	NA	6-Jun-09	14-Feb-09	-25.8	8.3
13538	Max	m	23	1999	na	na	15	3	2010	13-Apr-09	3-Apr-09	-25.5	7.7
13538	Max	m	23	1999	na	na	15	3	2010	13-Apr-09	12-Mar-09	-25.6	7.8
13538	Max	m	23	1999	na	na	15	3	2010	13-Apr-09	16-Feb-09	-25.8	7.8
13538	Max	m	23	1999	na	na	15	3	2010	13-Apr-09	19-Jan-09	-26.0	8.0
13538	Max	m	23	1999	na	na	15	3	2010	13-Apr-09	15-Dec-08	-26.3	8.1
13539	Pan	m	18	1998	na	na	15	3	2010	8-Jun-09	4-May-09	-25.3	8.2
13539	Pan	m	18	1998	na	na	15	3	2010	8-Jun-09	23-Feb-09	-25.4	8.1
13540	Tito	m	8	1989	na	na	7	7	2009	11-Apr-09	7-Mar-09	-25.5	8.5
13540	Tito	m	8	1989	na	na	7	7	2009	11-Apr-09	27-Dec-08	-25.6	8.4
13541	Tito	m	8	1989	na	na	7	7	2009	14-May-09	30-Apr-09	-25.4	8.5
13541	Tito	m	8	1989	na	na	7	7	2009	14-May-09	2-Apr-09	-25.7	8.5
13541	Tito	m	8	1989	na	na	7	7	2009	14-May-09	5-Mar-09	-25.9	8.5
13541	Tito	m	8	1989	na	na	7	7	2009	14-May-09	22-Jan-09	-26.0	8.5
13542	Tito	m	8	1989	na	na	7	7	2009	17-Jun-09	3-Jun-09	-25.3	8.6
13542	Tito	m	8	1989	na	na	7	7	2009	17-Jun-09	6-May-09	-25.5	8.5
13542	Tito	m	8	1989	na	na	7	7	2009	17-Jun-09	8-Apr-09	-25.7	8.4
13542	Tito	m	8	1989	na	na	7	7	2009	17-Jun-09	11-Mar-09	-25.9	8.4
13542	Tito	m	8	1989	na	na	7	7	2009	17-Jun-09	11-Feb-09	-26.1	8.3
