# Supplementary material for: The Steady State Great Ape? Long Term Isotopic Records Reveal the Effects of Season, Social Rank and Reproductive Status on Bonobo Feeding Behavior
Source: PLoS One. 2016 Sep 14;11(9):e0162091. doi: 10.1371/journal.pone.0162091 (PMC5023189; doi:10.1371/journal.pone.0162091)
Supplement: S2 Table — (PDF) [file pone.0162091.s004.pdf]

| species | family | tpy | ppu | food? | $\delta^{13}$C‰ vPDB | $\delta^{15}$N‰ AIR | %C | %N | atomic C:N | %protein (dry mass) | %protein (dry mass)* |
|---|---|---|---|---|---|---|---|---|---|---|---|
| *Haumania leonardiana* | *Marantaceae* | herb | pith | food | -33.7 | 4.9 | 49.5 | 3.4 | 16.8 | 21.4 | na |
| *Haumania liebrechtsiana* | *Marantaceae* | herb | leaf | food | -38.8 | 6.1 | 46.6 | 3.0 | 18.0 | 18.9 | 20.12 |
| *Haumania liebrechtsiana* | *Marantaceae* | herb | leaf | food | -33.7 | 6.4 | 46.6 | 3.0 | 18.1 | 18.8 | 20.12 |
| *Haumania liebrechtsiana* | *Marantaceae* | herb | pith | food | -33.2 | 6.4 | 43.5 | 6.0 | 8.5 | 37.2 | 30.74 |
| *Marantochloa purpurea* | *Marantaceae* | herb | leaves | food | -34.2 | 6.8 | 42.6 | 2.4 | 20.6 | 15.1 | 17.06 |
| *Sarcophrynium schweinfurth.* | *Marantaceae* | herb | leaves | food | -36.2 | 3.7 | 50.7 | 1.9 | 30.6 | 12.1 | 12.47 |
| *Unidentified* | *Unidentified* | herb | root | food | -28.7 | 3.0 | 49.3 | 1.1 | 52.9 | 6.8 | na |
| *Unidentified* | *Marantaceae* | herb | fruit | non-food | -31.0 | 5.6 | 42.1 | 1.4 | 34.3 | 8.9 | na |
| | | | | **mean** | **-33.7** | **5.4** | **46.4** | **2.8** | **25.0** | **17.4** | **20.1** |
| | | | | **1σ** | **3.0** | **1.4** | **3.3** | **1.5** | **13.9** | **9.5** | **6.7** |
| *Chytranthus gillettii* | *Sapindaceae* | shrub | seed | food | -32.3 | 2.8 | 43.7 | 1.6 | 31.4 | 10.2 | na |
| *Cola bruneelii* | *Sterculiaceae* | shrub | fruit | food | -37.8 | 5.9 | 45.3 | 3.0 | 17.4 | 19.0 | na |
| *Cola bruneelii* | *Sterculiaceae* | shrub | leaves | food | -33.3 | 5.1 | 47.0 | 3.4 | 16.3 | 21.1 | na |
| *Rothmannia macrocarpa* | *Rubiaceae* | shrub | fruit | non-food | -31.5 | 6.9 | 44.7 | 1.5 | 35.8 | 9.1 | na |
| | | | | **mean** | **-33.7** | **5.2** | **45.2** | **2.4** | **25.2** | **14.8** | **na** |
| | | | | **1σ** | **2.8** | **1.7** | **1.4** | **1.0** | **9.8** | **6.1** | **na** |
| *Cissus dinklagei* | *Vitaceae* | liana | fruit | food | -27.9 | 4.3 | 42.1 | 1.2 | 39.6 | 7.7 | 3.86 |
| *Cissus dinklagei* | *Vitaceae* | liana | fruit | food | -27.3 | 2.6 | 39.9 | 0.7 | 62.7 | 4.6 | na |
| *Dictyophleba ochracea* | *Apocynaceae* | liana | fruit | food | -28.4 | 6.0 | 47.2 | 1.5 | 36.9 | 9.3 | 5.11 |
| *Ficus sp.* | *Moraceae* | liana | fruit | food | -30.4 | 7.2 | 48.8 | 1.1 | 51.5 | 6.9 | 6.11 |
| *Ficus sp.* | *Moraceae* | liana | fruit | food | -32.5 | 5.8 | 50.1 | 1.1 | 51.2 | 7.1 | 6.11 |
| *Landolphia owariensis* | *Apocynaceae* | liana | fruit | food | -27.1 | 5.0 | 45.0 | 0.9 | 58.2 | 5.6 | 6.19 |
| *Manniophyton fulvum* | *Euphorbiaceae* | liana | leaves | food | -29.8 | 7.8 | 50.6 | 2.1 | 28.3 | 13.0 | na |
| *Manniophyton fulvum* | *Euphorbicaceae* | liana | leaves | medicine | -33.5 | 5.9 | 43.7 | 2.2 | 23.2 | 13.7 | na |
| *Manniophyton fulvum* | *Euphorbicaceae* | liana | leaves | medicine | -32.7 | 3.1 | 42.9 | 2.2 | 22.5 | 13.9 | na |
| *Manniophyton fulvum* | *Euphorbicaceae* | liana | leaves | medicine | -31.3 | 6.3 | 46.5 | 2.8 | 19.5 | 17.4 | na |
| | | | | **mean** | **-30.1** | **5.4** | **45.7** | **1.6** | **39.4** | **9.9** | **5.5** |
| | | | | **1σ** | **2.3** | **1.7** | **3.6** | **0.7** | **15.8** | **4.3** | **1.0** |
| *Blighia welwitschii* | *Sapindaceae* | tree | fruit | food | -28.6 | 5.3 | 71.6 | 2.5 | 33.6 | 15.5 | 10.16 |
| *Canarium schweinfurthii* | *Burseraceae* | tree | fruit | food | -29.3 | 6.1 | 62.2 | 0.9 | 77.6 | 5.8 | 6.03 |
| *Celtis brieyi* | *Ulmaceae* | tree | fruit | food | -29.2 | 3.2 | 46.2 | 2.4 | 22.2 | 15.2 | na |
| *Colletoecema dewevrei* | *Rubiaceae* | tree | fruit | food | -28.2 | 5.7 | 45.9 | 1.7 | 32.5 | 10.3 | na |
| *Dialium sp.* | *Caesalpiniaceae* | tree | fruit | food | -30.5 | 6.7 | 51.1 | 2.7 | 22.2 | 16.8 | 10.37 |
| *Dialium sp.* | *Caesalpiniaceae* | tree | fruit | food | -24.7 | 5.1 | 40.4 | 0.7 | 63.7 | 4.6 | 10.37 |
| *Drypetes cinnabarina* | *Euphorbiaceae* | tree | fruit | food | -28.7 | 6.1 | 47.2 | 1.2 | 44.5 | 7.7 | 4.66 |
| *Gambeya lacourtiana* | *Sapotaceae* | tree | fruit | food | -29.6 | 7.4 | 52.7 | 1.1 | 58.5 | 6.6 | 6.71 |
| *Gambeya lacourtiana* | *Sapotaceae* | tree | fruit | food | -25.7 | 5.1 | 58.4 | 1.5 | 44.4 | 9.6 | 6.71 |
| *Gambeya lacourtiana* | *Sapotaceae* | tree | fruit | food | -28.4 | 6.6 | 55.9 | 1.1 | 61.4 | 6.6 | 6.71 |
| *Garcinia punctata* | *Clusiaceae* | tree | fruit | food | -29.1 | 6.2 | 49.0 | 1.2 | 47.0 | 7.6 | 5.9 |
| *Garcinia punctata* | *Clusiaceae* | tree | fruit | food | -31.6 | 6.1 | 54.8 | 1.0 | 64.4 | 6.2 | na |
| *Grewia coriacea* | *Tiliaceae* | tree | fruit | food | -28.2 | 4.9 | 42.4 | 1.0 | 47.9 | 6.5 | 14.99 |
| *Irvingia gabonensis* | *Irvingiaceae* | tree | fruit | food | -26.9 | 6.0 | 43.9 | 0.9 | 55.0 | 5.8 | na |
| *Irvingia sp.* | *Irvingiaceae* | tree | fruit | food | -26.4 | 6.6 | 45.6 | 1.5 | 36.6 | 9.1 | 6.83 |
| *Klainedoxa gabonensis* | *Irvingiaceae* | tree | fruit | food | -33.4 | 5.4 | 43.9 | 1.1 | 47.4 | 6.8 | 2.3 |
| *Mammea africana* | *Clusiaceae* | tree | fruit | food | -27.7 | 4.6 | 47.5 | 0.6 | 87.4 | 4.0 | 4.83 |
| *Manilkara yangambiensis* | *Sapotaceae* | tree | fruit | food | -27.1 | 4.6 | 45.2 | 0.9 | 57.3 | 5.8 | 4.71 |
| *Nauclea sp.* | *Rubiaceae* | tree | fruit | food | -28.1 | 7.0 | 45.9 | 0.4 | 124.4 | 2.7 | na |
| *Pancovia laurentii* | *Sapindaceae* | tree | fruit | food | -28.2 | 6.1 | 45.9 | 2.5 | 21.1 | 15.9 | 7.13 |
| *Pancovia laurentii* | *Sapindaceae* | tree | fruit | food | -27.1 | 5.7 | 43.7 | 1.1 | 44.5 | 7.2 | 7.13 |
| *Parinari excelsa* | *Chrysobalanaceae* | tree | fruit | food | -28.5 | 5.7 | 46.9 | 0.8 | 65.2 | 5.2 | 4.8 |
| *Phyllanthus pynaertii* | *Euphorbiaceae* | tree | fruit | food | -29.9 | 2.5 | 44.1 | 1.2 | 43.1 | 7.5 | na |
| *Scorodophloeus zenkeri* | *Caesalpiniaceae* | tree | fruit | food | -26.6 | 7.0 | 46.3 | 3.6 | 14.9 | 22.6 | 16.94 |
| *Sorindea sp.* | *Anacardiaceae* | tree | fruit | food | -28.9 | 6.9 | 47.7 | 1.3 | 44.3 | 7.8 | 7.83 |
| *Synsepalum sp.* | *Sapotaceae* | tree | fruit | food | -26.2 | 3.5 | 41.8 | 1.6 | 30.8 | 9.9 | 11.28 |
| *Tessmannia africana* | *Caesalpiniaceae* | tree | fruit | food | -27.1 | 7.6 | 43.0 | 2.5 | 20.1 | 15.6 | na |
| *Trichoscypha arborescens* | *Anacardiaceae* | tree | fruit | food | -27.2 | 6.6 | 49.5 | 1.2 | 47.2 | 7.7 | 4.16 |
| *Trichoscypha arborescens* | *Anacardiaceae* | tree | fruit | food | -27.8 | 4.7 | 42.8 | 1.5 | 33.8 | 9.2 | na |
| *Unidentified* | *Vitaceae* | tree | fruit | food | -27.8 | 6.5 | 52.9 | 1.0 | 62.3 | 6.2 | 7.51 |
| *Camptostylus mannii* | *Flacourtiaceae* | tree | fruit | non-food | -34.2 | 7.5 | 51.2 | 2.3 | 26.1 | 14.3 | na |
| *Drypetes cinnabarina* | *Euphorbiaceae* | tree | fruit | non-food | -26.9 | 5.3 | 48.8 | 1.1 | 49.5 | 7.2 | 9.3 |
| *Onkogea gore* | *Olacaceae* | tree | fruit | non-food | -28.3 | 5.6 | 45.4 | 2.1 | 25.6 | 12.9 | 13.99 |
| *Picralima nitida* | *Apocynaceae* | tree | fruit | non-food | -32.2 | 9.3 | 44.3 | 1.4 | 35.7 | 9.0 | na |
| | | | | **tree fruit mean** | **-28.5** | **5.9** | **48.4** | **1.5** | **46.8** | **9.2** | **8.0** |
| | | | | **1σ** | **2.1** | **1.3** | **6.4** | **0.7** | **21.9** | **4.4** | **3.6** |
| *Cynometra sessiliflora* | *Caesalpiniaceae* | tree | leaves | food | -29.6 | 7.1 | 50.8 | 2.7 | 22.1 | 16.8 | na |
| *Gilbertiodendron dewevrei* | *Caesalpiniaceae* | tree | seed | food | -28.2 | 6.1 | 42.8 | 0.9 | 58.1 | 5.4 | 6.04 |
| *Phyllanthus pynaertii* | *Euphorbiaceae* | tree | stem | food | -32.0 | 3.9 | 42.6 | 3.4 | 14.6 | 21.2 | 19.93 |
| *Phyllanthus pynaertii* | *Euphorbiaceae* | tree | stem | food | -30.4 | 4.1 | 42.4 | 2.1 | 23.4 | 13.2 | 19.93 |
| *Scorodophloeus zenkeri* | *Caesalpiniaceae* | tree | leaf | food | -31.1 | 8.3 | 46.6 | 5.2 | 10.5 | 32.2 | 20.21 |
| *Scorodophloeus zenkeri* | *Caesalpiniaceae* | tree | leaves | food | -33.4 | 8.3 | 40.9 | 2.0 | 23.5 | 12.7 | 20.21 |
| *Scorodophloeus zenkeri* | *Caesalpiniaceae* | tree | leaves | food | -33.4 | 8.3 | 41.6 | 3.5 | 14.0 | 21.7 | 20.21 |
| *Scorodophloeus zenkeri* | *Caesalpiniaceae* | tree | seed | food | -26.7 | 7.4 | 41.1 | 3.2 | 15.0 | 20.0 | 16.94 |
| *Thonnera congolana* | *Annonaceae* | tree | seed | non-food | -32.5 | 7.9 | 54.8 | 2.2 | 29.3 | 13.7 | 7.45 |
| *Cleistopholis sp.* | *Annonaceae* | tree | bark | non-food | -28.8 | 3.2 | 49.9 | 0.6 | 97.6 | 3.7 | na |
| *Dicranolepis soyauxii* | *Thymeleaceae* | tree | bark | non-food | -29.4 | 5.1 | 52.8 | 0.5 | 113.8 | 3.4 | na |
| *Dicranolepis soyauxii* | *Thymeleaceae* | tree | bark | non-food | -29.6 | 4.7 | 54.6 | 0.6 | 108.1 | 3.7 | na |
| *Pancovia laurentii* | *Sapindaceae* | tree | seed | non-food | -28.5 | 6.8 | 46.4 | 2.3 | 23.7 | 14.2 | 9.88 |
| *Unidentified* | *Unidentified* | tree | bark | non-food | -26.9 | 4.3 | 47.2 | 1.2 | 47.6 | 7.2 | na |
| | | | | **tree non-fruit mean** | **-30.0** | **6.1** | **46.7** | **2.2** | **43.0** | **13.5** | **15.6** |
| | | | | **1σ** | **2.2** | **1.8** | **5.1** | **1.4** | **36.9** | **8.5** | **6.1** |
